# ENHO and EDN1 variants link adropin–endothelin-1 imbalance with early vascular aging in adults with and without type 2 diabetes

**DOI:** 10.3389/fendo.2026.1961984

**Published:** 2026-10-06

**Authors:** Rooban Sivakumar, K. A. Arul Senghor, V. M. Vinodhini, Janardhanan Kumar

**Affiliations:** 1 Department of Biochemistry, SRM Medical College Hospital and Research Centre, Faculty of Medicine and Health Sciences, SRM Institute of Science and Technology, SRM Nagar, Kattankulathur, Tamil Nadu, India; 2 Department of General Medicine, SRM Medical College Hospital and Research Centre, Faculty of Medicine and Health Sciences, SRM Institute of Science and Technology, Kattankulathur, Tamil Nadu, India

**Keywords:** EDN1, ENHO, genetic risk score, SNP, type 2 diabetes mellitus

## Abstract

**Background:**

Early vascular aging (EVA) is common in type 2 diabetes, yet its inherited determinants remain undefined. This study examined the associations of ENHO and EDN1 genetic variation with circulating adropin, endothelin-1, their relative imbalance, and early vascular aging in adults with and without T2DM.

**Methods:**

This study included 300 adults aged 25–55 years, comprising 150 participants with T2DM and 150 age- and sex-matched controls. Participants were further classified as having Early vascular aging (EVA) (n=116) or Normal vascular aging (NVA) (n=184) using age-referenced estimated pulse wave velocity. ENHO rs2281997 and rs72735260, and *EDN1* rs5370 and rs2070699 were genotyped by HRM analysis with Sanger confirmation. Gene-specific and total genetic risk scores (GRS) were tested against adropin, endothelin-1, the adropin–endothelin-1 imbalance index (AEI), EVA, and continuous ePWV.

**Results:**

In the overall cohort, all four variants were associated with EVA in the risk direction. rs5370 and rs72735260 remained significant after FDR correction. Each additional risk allele in the Total GRS increased the adjusted odds of EVA by 69% (OR 1.691, 95% CI 1.381–2.094), with a graded increase across genetic burden categories. Higher ENHO-GRS was associated with lower adropin, higher EDN1-GRS with higher endothelin-1, and higher Total GRS with greater AEI, which statistically mediated 51.8% of the GRS–EVA association. Within the study cohort, AEI discriminated EVA better than GRS (AUC 0.776 vs 0.661).

**Conclusions:**

Combined *ENHO*–*EDN1* genetic burden is associated with an adverse adropin–endothelin-1 balance and early vascular aging. Independent replication is required before clinical application.

## 1 Introduction

Type 2 diabetes mellitus (T2DM) is associated with an approximately two- to fourfold increase in cardiovascular risk, yet conventional risk factors explain only about one-third of this excess burden (1, 2). Chronic hyperglycemia and insulin resistance reduce endothelial nitric oxide bioavailability, enhance endothelin-1-mediated vasoconstrictive signaling, and promote collagen accumulation, advanced glycation, and vascular calcification (3, 4). The resulting structural and functional changes progressively increase arterial stiffness, often before cardiovascular disease becomes clinically apparent. Pulse wave velocity is therefore elevated even in individuals with T2DM who do not have established macrovascular complications (5, 6). Early vascular aging (EVA) describes a mismatch between chronological age and the biological condition of the large arteries and has been independently associated with cardiovascular events and mortality (7, 8). Estimated pulse wave velocity (ePWV), calculated from age and mean blood pressure, provides a practical measure of this vascular phenotype (9, 10). Age-referenced interpretation is especially useful in middle-aged adults because it helps separate pathological arterial stiffening from the increase expected during normal aging (11). Premature vascular aging in T2DM may therefore reflect the combined influence of cumulative metabolic injury, disturbed endothelial signaling, and inherited biological susceptibility.

Adropin is a 76-amino-acid secreted peptide encoded by the energy homeostasis-associated gene, ENHO, located on chromosome 9p13.3. It is expressed in the liver, brain, heart, and vascular endothelium (12). Adropin promotes a vasoprotective endothelial phenotype by upregulating VEGFR-2 expression and activating PI3K/Akt and ERK1/2 pathways. Both pathways lead to phosphorylation of endothelial nitric oxide synthase at Ser1177 and increased nitric oxide bioavailability (13, 14). This mechanism is consistent with previous reports showing the restoration of endothelium-dependent vasodilation by adropin in aged human arteries and the attenuation of the vascular protective effects of adropin in eNOS-deficient models (15, 16). The vascular effects of endothelin-1 are predominantly opposite. Activation of ETA receptors on vascular smooth muscle cells leads to vasoconstriction, cellular proliferation, fibrosis and inflammation, while endothelial ETB receptors mediate nitric oxide-dependent vasodilation and ET-1 clearance (17, 18). Nitric oxide can suppress EDN1 transcription, while prolonged ET-1 exposure may reduce eNOS expression (19). Nitric oxide bioavailability may therefore act as a shared regulatory point linking the adropin and ET-1 pathways rather than the two peptides functioning as unrelated circulating markers.

Several studies support this biological connection with arterial stiffness. Lower circulating adropin has been associated with greater carotid β-stiffness index in middle-aged and older adults, while exercise-related increases in adropin have occurred alongside reductions in arterial stiffness and increases in nitric oxide metabolites (20). By contrast, circulating ET-1 has independently predicted carotid-femoral pulse wave velocity, and selective ETA-receptor blockade has reduced pulse wave velocity in a randomized crossover trial (21, 22). Studies measuring both peptides in the same participants have also reported lower adropin and higher ET-1 in the hypertensive group (23) and in patients with obstructive sleep apnea–hypopnea syndrome (OSAHS) (24), together with an inverse relationship between their circulating concentrations. This opposing pattern supports assessment of their relative balance rather than interpretation of either peptide alone. Similar ratio-based measures are already used in vascular biology, including the arginine-to-asymmetric dimethylarginine and angiotensin-(1-7)-to-angiotensin II indices, which may provide greater biological information than their individual components (25, 26). In the previously characterized cohort, lower adropin and higher ET-1 were independently associated with early vascular aging, and their combined assessment discriminated EVA better than either marker alone (27). The extent to which this adverse peptide balance is influenced by inherited variation in ENHO and EDN1 has not been established.

Genetic variation provides a plausible upstream source of differences within this peptide axis. Plasma ET-1 has an estimated heritability of approximately 58%, while a genome-wide analysis of a stable ET-1 surrogate attributed 14.6% of its phenotypic variance to common genetic variants and identified EDN1 rs5370 among the leading signals (28, 29). Comparable heritability estimates are not available for adropin, although variation within ENHO has been associated with circulating adropin concentrations (30). Among the variants examined in the present study, ENHO rs2281997 has been linked to adropin concentrations and dyslipidemia (30, 31). The rs72735260 variant lies within the same DNase I hypersensitive region and may have regulatory relevance, although evidence for an independent phenotypic effect remains limited (30, 31). Within EDN1, the missense variant rs5370 has been associated with blood pressure in individuals with overweight and with coronary artery disease, but these findings have not been reproduced consistently (32–34). The noncoding rs2070699 T allele has also been associated with higher circulating ET-1 and adverse vascular outcomes (35). Allele frequencies and linkage disequilibrium patterns vary across ancestral populations, and these loci have received limited study in Indian populations. Their relevance therefore requires direct evaluation in South Asian cohorts (36, 37).

Current evidence has not connected these genetic, biochemical, and vascular levels within the same individuals (30, 31). ENHO variants have been studied in relation to circulating adropin without concurrent assessment of vascular aging, while EDN1 variants have generally been examined against clinical outcomes without measurement of circulating ET-1. Individual common variants usually have small phenotypic effects, making pathway-specific genetic scores potentially more informative than isolated single-variant analysis for representing cumulative inherited susceptibility (38, 39). Integrated genetic and biomarker models have also shown value in cardiovascular risk assessment (40, 41). However, it remains unknown whether combined ENHO–EDN1 variation is reflected in an adverse adropin–ET-1 balance and whether this pathway-level pattern is associated with premature vascular aging. To address this gap, the present study examined genetic, biochemical, and vascular relationships in a cohort of adults with and without T2DM using a prespecified analytical hierarchy. ENHO rs2281997 and rs72735260 and EDN1 rs5370 and rs2070699 were evaluated individually and subsequently combined into gene-specific genetic risk scores (ENHO-GRS and EDN1-GRS) and a total pathway score (Total GRS). Their associations with circulating adropin, ET-1, the adropin–endothelin-1 imbalance index, early vascular aging, and continuous ePWV were then assessed.

## 2 Materials and methods

### 2.1 Study design, setting, and participants

This genetic association study was carried out at SRM Medical College Hospital and Research Centre, SRM Institute of Science and Technology, Kattankulathur, Tamil Nadu, India, from July 2024 to October 2025. The analysis draws on a previously characterized observational cohort of 300 adults aged 25 to 55 years, in which 150 individuals with type 2 diabetes mellitus (T2DM) were enrolled alongside 150 normoglycemic control participants matched for age and sex (27). Participants in the T2DM group required a physician-confirmed diagnosis of at least two years’ duration. Controls were healthy adults without diabetes, defined as HbA1c <5.7% and fasting plasma glucose <100 mg/dL (42). They had no history of chronic disease, recent acute illness, cardiovascular disease, or long-term medication use. Participants with established cardiovascular disease, chronic kidney disease, autoimmune disease, or a current pregnancy, or if hospitalization, febrile illness, or surgery had occurred in the 45 days preceding assessment, were excluded from this study. Ethical clearance was granted by the Institutional Ethics Committee of SRM Medical College Hospital and Research Centre (approval no. ECR/8856/INST/TN/2013/RR-19). Every participant provided written informed consent before enrollment, and all procedures conformed to the principles of the Declaration of Helsinki. The cohort, the age-referenced ePWV-based EVA classification, and the serum adropin and endothelin-1 measurements have been reported previously (27). The present study is a distinct analysis of the same participants and adds genotyping of *ENHO* and *EDN1* variants, genetic risk score construction, haplotype and linkage-disequilibrium analysis, and mediation modeling. Written consent obtained at enrollment covered subsequent genetic analysis of stored samples. Statistical power for the genetic association analysis was evaluated using Quanto under a dominant model and Power for Genetic Association under a codominant model. At a two-sided α of 0.05, the available sample of 300 participants was sufficient to detect moderate-to-strong variant–phenotype associations (43).

### 2.2 Clinical and anthropometric assessment

A detailed proforma sheet was used to capture age, sex, duration of diabetes, current medications, smoking and alcohol use, and relevant past medical history. Standing height and body weight were obtained with calibrated instruments, and body mass index was calculated. Brachial blood pressure was recorded using an oscillometric device incorporating atrial fibrillation detection (Omron HEM-7361T; Omron Healthcare Co., Ltd., Kyoto, Japan). Participants rested quietly in the seated position for at least five minutes, after which three sequential readings were recorded at 30-second intervals and averaged. Mean blood pressure (MBP) was derived as:

MBP = DBP + 0.4 × (SBP − DBP), where SBP and DBP denote systolic and diastolic blood pressure (44).

### 2.3 Biochemical and biomarker measurements

Venous blood was drawn following an overnight fast of 8 to 10 hours into appropriate vacutainer tubes, and a further post-prandial sample was obtained for post-prandial plasma glucose. Fasting plasma glucose and the lipid panel were quantified on a Beckman Coulter DxC 700 automated clinical chemistry analyzer (Beckman Coulter, Brea, CA, USA). Glycated hemoglobin was determined by high-performance liquid chromatography on a Bio-Rad D-10 system (Bio-Rad Laboratories, Hercules, CA, USA). Enzymatic CHOD–POD and GPO–POD chemistries were applied to total cholesterol and triglycerides, respectively, while HDL-C and LDL-C were assayed by direct antibody-inhibition methods. Remaining serum was separated, aliquoted, and held at −80 °C until biomarker quantification. Serum adropin (Cat. OPK6444) and endothelin-1 (Cat. OPK2444-M) were measured by commercial sandwich enzyme-linked immunosorbent assay (Origin Diagnostics & Research, Kerala, India). Absorbance was measured using a Bio-Rad PR 4100 microplate reader, and analyte concentrations were interpolated from assay-specific standard curves using the mean optical density of duplicate wells. All procedures were performed following manufacturer’s instructions. The analytical sensitivity and measuring range declared by the manufacturer were 14.2 pg/mL and 31.25 to 2000 pg/mL for adropin and 0.75 pg/mL and 1.25 to 80 pg/mL for endothelin-1. The intra-assay and inter-assay coefficients of variation reported were <8% and <10%, respectively.

### 2.4 Vascular aging phenotype and the adropin–endothelin-1 imbalance index

Estimated pulse wave velocity was computed from age and MBP using the published regression equation appropriate to the presence or absence of cardiovascular risk factors (44, 45). Early vascular aging was defined using the age-referenced, residual-based ePWV method developed and reported in our previous study (27). The same predefined classification was applied in the present analysis. Binary EVA status was the primary vascular outcome, while continuous ePWV was analyzed as a secondary outcome to assess genetic associations across the full spectrum of arterial stiffness. For the present study, the adropin–endothelin-1 imbalance index (AEI) was constructed from the standardized log-transformed concentrations of the two peptides to capture their opposing vascular profiles, as supported by previous studies reporting reciprocal changes in circulating adropin and ET-1 (23, 24, 27):

AEI = z[ln(ET-1)] − z[ln(adropin)]

Higher AEI values therefore denote relative endothelin-1 predominance in the setting of lower adropin.

### 2.5 Candidate variant selection

A pathway-restricted candidate-gene strategy was used to evaluate ENHO, which encodes adropin, and EDN1, which encodes endothelin-1. Variants located within or adjacent to these loci were retrieved from NCBI dbSNP (https://www.ncbi.nlm.nih.gov/snp/) and initially restricted to those with a minor allele frequency >0.05 to ensure adequate genotype representation for association testing. Merged or old dbSNP records were mapped to their current reference identifiers, duplicate entries were removed, and the remaining records were limited to single-nucleotide variants. Likewise, deletions, insertion–deletions, and other complex variant classes were excluded. Common SNVs were then prioritized according to prespecified criteria, including genomic location, coding or regulatory context, biological relevance to the encoded peptide, previously reported biomarker or cardiometabolic associations, and availability of interpretable population-frequency data. The presence of all shortlisted variants in the Indian population was confirmed using the IndiGenomes database (https://clingen.igib.res.in/indigen/). The detailed selection process is explained in **Supplementary Table S1**.

### 2.6 DNA extraction, high-resolution melting genotyping and sanger sequencing

Genomic DNA was isolated from peripheral blood using the Origin Genomic DNA Kit (Cat no. ODP301). DNA concentration and purity were assessed using a NanoDrop spectrophotometer. Samples with an A260/A280 ratio of approximately 1.8 and an A260/A230 ratio between 2.0 and 2.2 were considered acceptable and carried forward for genotyping. Extracted DNA was stored at -80 °C until analysis. All variants were genotyped by high-resolution melting (HRM) analysis on a Bio-Rad CFX96 real-time PCR platform. Locus-specific primers were custom synthesized by Eurofins Genomics; specificity was checked in silico by NCBI BLAST, and every assay was designed to yield a short amplicon of < 150 bp, which is suitable for HRM-based allelic discrimination. Primer sequences, amplicon lengths, and melting-temperature separations are provided in **Supplementary Table S2**.

The final reaction volume of 20 µL was made up using Origin 2× HRM Master Mix (Cat. No. OHRM-M), which supplies a hot-start DNA polymerase together with a proprietary saturating double-stranded DNA-binding dye. Each reaction contained 10 µL of the 2× master mix, 1 µL each of forward and reverse primers at 10 pmol/µL, 50 ng/μL genomic DNA, and nuclease-free water to volume. Cycling comprised hot-start activation at 95 °C for 12 min, then 40 cycles of 95 °C for 15 s, 63 °C for 20 s, and 72 °C for 20 s. Melting was performed immediately afterwards from 65 °C to 95 °C at a ramp rate of 0.1 °C/s, with fluorescence captured on the SYBR/FAM channel. Amplification and melt data were processed in Bio-Rad Precision Melt Analysis software v1.2. Normalized melt curves, derivative plots, and difference plots were reviewed together during genotype assignment. Representative amplicons drawn randomly from each HRM-defined genotype cluster were sequenced on a SeqStudio Genetic Analyzer (Applied Biosystems, Waltham, MA, USA). Chromatograms were inspected in Unipro UGENE V53.1 (Unipro UGENE Team, Novosibirsk State University, Russia) (46).

### 2.7 Genetic modeling and risk scores

Genotype and allele frequencies were summarized for the full cohort and by vascular-aging status. The T allele was modeled as the risk allele for all four variants. Hardy–Weinberg equilibrium was assessed by exact testing in the NVA group, with the EVA group examined as a sensitivity analysis. Each SNP was evaluated under codominant, dominant, recessive, over-dominant, and log-additive models. The log-additive model coded genotypes as 0, 1, or 2 according to T-allele dosage. Unweighted genetic risk scores were calculated as:

ENHO-GRS= rs2281997_T_ + rs72735260_T_

EDN1-GRS= rs5370_T_ + rs2070699_T_

Total GRS = ENHO-GRS + EDN1-GRS

The Total GRS ranged from 0 to 8 risk alleles and was analyzed continuously and as low (0–1), intermediate (2), and high (≥3) genetic burden.

### 2.8 Linkage disequilibrium, haplotypes, and in silico annotation

Pairwise linkage disequilibrium was quantified using D′, which reflects historical recombination between the variants, and r², which reflects the correlation between alleles and therefore how well one variant predicts the other. Gene-specific haplotypes were reconstructed for *ENHO* rs2281997–rs72735260 and *EDN1* rs5370–rs2070699, and haplotype frequencies, global association tests, and haplotype-specific estimates were evaluated as secondary analysis. In silico annotation was performed after variant selection had been finalized. Genomic coordinates (GRCh38) and molecular consequence were obtained from dbSNP and associated annotation resources; ClinVar (https://www.ncbi.nlm.nih.gov/clinvar/) was queried for reported clinical classifications, RegulomeDB (https://regulomedb.org/regulome-search/) for regulatory evidence, and SpliceAI (https://spliceailookup.broadinstitute.org/) for predicted splicing effects where relevant.

### 2.9 Statistical analysis

Continuous variables were assessed for distributional characteristics using visual inspection of histograms and Q–Q plots, with the Kolmogorov-Smirnov test used as a supplementary assessment of normality. Normally distributed continuous variables were summarized as mean ± standard deviation, skewed variables as median with interquartile range, and categorical variables as frequency and percentage. Between-group comparisons used the independent-samples *t* test or Mann–Whitney U test for continuous variables and the chi-square or Fisher exact test for categorical variables, as appropriate. Analyses were performed in R version 4.4.2 using the stats package for regression and group comparisons, HardyWeinberg package for Hardy–Weinberg equilibrium testing, haplo.stats for haplotype analysis, mediation for mediation analysis, and pROC for ROC analysis. All tests were two-sided, with *p* < 0.05 considered statistically significant. Benjamini–Hochberg false-discovery-rate (FDR) correction was applied within prespecified families of related tests. For the alternative inheritance-model analysis of EVA and continuous ePWV, FDR correction was applied separately within each SNP across the six inheritance-model contrasts. For genotype–biomarker analysis, FDR correction was applied separately for each biomarker outcome across the four SNP allele-dosage tests. Gene-specific and Total GRS analysis were prespecified pathway-level analysis and were therefore reported with nominal p values rather than included in the SNP-level FDR families. Associations between individual SNPs and binary EVA status were examined by logistic regression under codominant, dominant, recessive, over-dominant, and log-additive inheritance models. Results were reported as odds ratios with 95% confidence intervals. Model fit was compared using the Akaike information criterion. Associations with continuous ePWV were evaluated using corresponding linear-regression models. Multivariable logistic and linear regression models were adjusted for age, sex, BMI, and HbA1c. There were no missing values for variables included in the primary regression analysis, therefore no imputation was required. Following this, ENHO-GRS, EDN1-GRS, and Total GRS were tested against EVA using logistic regression and against continuous ePWV using linear regression. Total GRS was analyzed continuously and by low, intermediate, and high genetic-burden categories. Mediation analysis evaluated AEI as a statistical mediator of the association between Total GRS and EVA, with continuous ePWV examined as a parallel outcome. Indirect, direct, and total effects and the proportion mediated were estimated using nonparametric bootstrap resampling with 5,000 iterations and 95% confidence intervals. Given the cross-sectional design, mediation findings were interpreted as pathway-consistent associations rather than evidence of causality. Exploratory ROC analysis were conducted to assess within-cohort discrimination of EVA. Areas under the curve were reported with 95% confidence intervals and compared using DeLong’s test. Because no independent external validation cohort was available, the ROC results were interpreted as exploratory within-cohort discrimination rather than validated predictive performance.

## 3 Results

### 3.1 Study population and vascular-aging phenotype

The analysis included 300 participants, comprising 150 normoglycemic controls and 150 participants with T2DM. Based on age-referenced ePWV, 184 participants were classified as having normal vascular aging (NVA) and 116 as having early vascular aging (EVA). The clinical, biochemical, and vascular characteristics of the cohort are summarized in **Table 1**. As expected, participants with T2DM had higher fasting plasma glucose (132.5 vs 91 mg/dL; *p* < 0.001), post-prandial plasma glucose (203 vs 116 mg/dL; *p* < 0.001), HbA1c (7.4% vs 5.2%; *p* < 0.001), and estimated average glucose (166 vs 103 mg/dL; *p* < 0.001), when compared with controls. They also had higher total cholesterol, triglycerides, LDL-C, VLDL-C, non-HDL-C, and atherogenic lipid ratios, along with lower HDL-C (43.5 vs 46 mg/dL; *p* = 0.021). Median ePWV was higher in T2DM than in controls (8.02 vs 7.21 m/s; *p* < 0.001), whereas adropin was lower (245.27 vs 834.24 pg/mL; *p* < 0.001) and ET-1 was higher (5.59 vs 1.29 pg/mL; *p* < 0.001) (**Table 1**).

**Table 1:** Clinical and biochemical characteristics of the study population.

Parameter	Control (n=150)	T2DM (n=150)	p^a^- value	Normal VA (n=184)	Early VA (n=116)	p^b^-value
Age, years	48 (43,52)	49 (45,53)	0.185	47 (43,52)	50 (45,53)	0.062
Male, n (%)	79 (52.6)	73 (48.6)	0.488	95 (51.6)	58 (50.0)	0.783
BMI, kg/m²	22.21 (21.5,22.8)	24.38 (22.3,26.2)	**<0.001**	22.48 (21.55,23.66)	23.31 (22.07,25.24)	**0.004**
SBP, mmHg	116 (112,120)	126 (120,132)	**<0.001**	117 (113,121)	128 (122,135)	**<0.001**
DBP, mmHg	74 (71,76)	80 (76,88)	**<0.001**	73 (72,77)	82 (77,90)	**<0.001**
FPG, mg/dL	91 (86,95)	132.5 (110,176)	**< 0.001**	95 (88,103.5)	126 (100,166)	**<0.001**
PPPG, mg/dL	116 (110,123.25)	203 (151,254)	**< 0.001**	121 (113,139.5)	198 (127,249)	**<0.001**
HbA1c, %	5.2 (5,5.4)	7.4 (6.6,9.3)	**< 0.001**	5.4 (5.1, 6.2)	7.4 (6.5,9.4)	**<0.001**
eAG	103 (97,108)	166 (142.5,220)	**< 0.001**	108 (100,131)	166 (129,223)	**<0.001**
Total cholesterol, mg/dL	154 (130,181.5)	180 (161.75,216)	**< 0.001**	166 (138.5,185.5)	178 (153,216)	**<0.001**
Triglycerides, mg/dL	97 (71,123.75)	120.5 (95,163.5)	**< 0.001**	100 (76,133)	122 (91,165)	**<0.001**
HDL-C, mg/dL	46 (41,52)	43.5 (38,49)	**0.021**	46 (41,52)	43 (37,49)	**0.012**
LDL-C, mg/dL	112.5 (91,128.5)	124 (107,148.5)	**< 0.001**	117 (95,132)	120 (105,150)	**0.040**
VLDL-C, mg/dL	19 (14,25)	24 (19,33)	**< 0.001**	20 (15,27)	24 (18,33)	**<0.001**
Non-HDL-C, mg/dL	115 (93.25,133)	134 (115,163)	**< 0.001**	121 (96.25,139)	131 (111,160.5)	**<0.001**
TC/HDL-C	3.56 ± 0.82	4.19 ± 0.98	**< 0.001**	3.67 ± 0.88	4.1 ± 0.99	**<0.001**
LDL-C/HDL-C	2.49 ± 0.73	2.93 ± 0.80	**< 0.001**	2.57 ± 0.78	2.90 ± 0.79	**<0.001**
TGL/HDL-C	2.4 ± 1.03	3.29 ± 1.7	**< 0.001**	2.50 ± 1.30	3.34 ± 1.25	**<0.001**
ePWV, m/s	7.21 (6.86,7.7)	8.02 (7.39,8.49)	**< 0.001**	7.17 (6.85,7.44)	8.18 (7.91, 8.63)	**<0.001**
Adropin, pg/mL	834.24 (653.4, 1884.9)	245.27 (119.85,528.74)	**< 0.001**	867.21 (447, 1873.55)	227 (113,297.17)	**<0.001**
ET-1, pg/mL	1.29 (1.16,1.43)	5.59 (3.03,7.78)	**< 0.001**	1.42 (1.24,2.7)	5.2 (2.1,7.2)	**<0.001**

Data are presented as median (interquartile range) or mean ± standard deviation. p^a^, control vs T2DM; p^b^, NVA vs EVA. FPG, fasting plasma glucose; PPPG, post-prandial plasma glucose; eAG, estimated average glucose; HDL-C, high-density lipoprotein cholesterol; LDL-C, low-density lipoprotein cholesterol; VLDL-C, very-low-density lipoprotein cholesterol; ePWV, estimated pulse wave velocity; ET-1, endothelin-1. Bold values indicate statistical significance at p < 0.05.

Following this, participants with EVA had higher fasting glucose (126 vs 95 mg/dL; *p* < 0.001), post-prandial glucose (198 vs 121 mg/dL; *p* < 0.001), HbA1c (7.4% vs 5.4%; *p* < 0.001), and estimated average glucose (166 vs 108 mg/dL; *p* < 0.001). Total cholesterol, triglycerides, VLDL-C, non-HDL-C, and atherogenic lipid ratios were higher in EVA with lower HDL-C levels (43 vs 46 mg/dL; *p* = 0.012). Similarly, decreased levels of adropin (227 vs 867.21 pg/mL; *p* < 0.001) were observed in EVA with increased ET-1 levels (5.20 vs 1.42 pg/mL; *p* < 0.001) when compared to NVA (**Table 1**).

### 3.2 Candidate-variant selection and final SNP panel

The candidate-variant screening process began with 19,195 annotated records, comprising 2,095 ENHO variants and 17,100 EDN1 variants. Limiting the analysis to common variants with a minor allele frequency greater than 0.05 reduced this set to 243 records, including 5 ENHO and 238 EDN1 variants. After resolving merged and deprecated rs identifiers, 101 unique current variants remained. Non-SNV and complex records were removed, leaving a set of 85 unique common SNVs. These variants were then prioritized according to functional consequence, interpretable gene context, and supporting evidence from public databases and the literature, yielding a shortlist of 10 candidates. All shortlisted variants were confirmed in the IndiGenomes Indian-frequency dataset. From this set, a final pathway-balanced panel of four variants was selected: ENHO rs2281997 and rs72735260, and EDN1 rs5370 and rs2070699 (**Figure 1**; **Supplementary Table S1****).**

**Figure 1:**
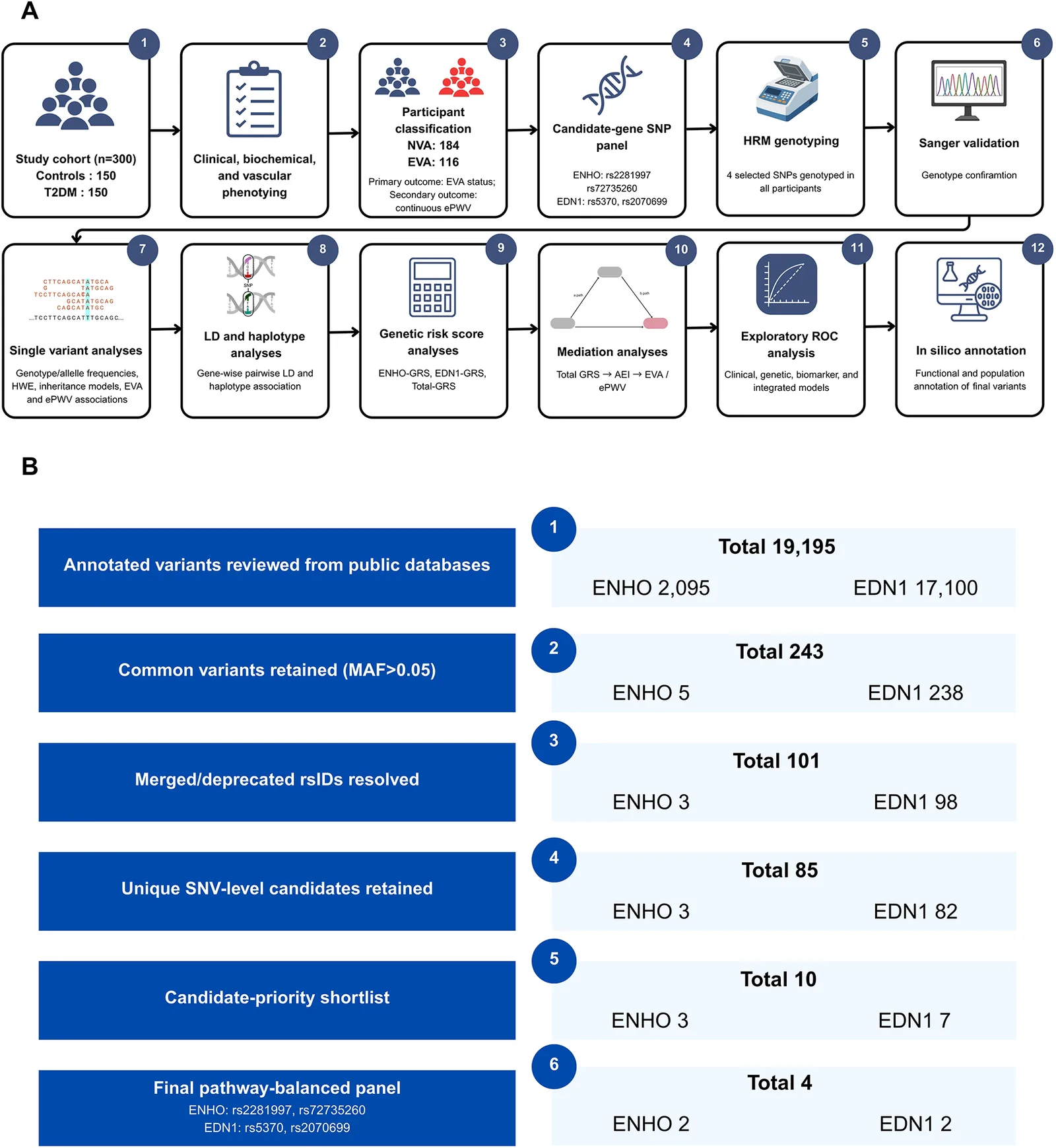
Study workflow and candidate-variant selection. **(A)** Overview of the analytical workflow. **(B)** Evidence-prioritization funnel used for candidate-variant selection.

### 3.3 HRM assay performance and Sanger validation

All four variants were genotyped using the HRM assay. Assay-specific details, including primer sequences, amplicon sizes, melting-temperature separation, and in silico specificity assessment, are provided in **Supplementary Table S2**. Each variant was run in replicates, producing distinct and reproducible HRM melting profiles corresponding to the different genotype patterns. Multiple representative samples from each HRM-defined genotype group were subsequently validated by Sanger sequencing, confirming the genotype assignments obtained by HRM (**Supplementary Figure S1**).

### 3.4 Genotype distribution and allele frequency

Minor-allele frequencies differed consistently between the vascular-aging groups. The T allele was more frequent in participants with EVA than in those with NVA for each of the four variants. Genotype distributions in the NVA reference group showed no significant deviation from Hardy-Weinberg equilibrium, with *p* values > 0.05. Minor-homozygote counts were low for the two ENHO variants, with only 11 participants carrying the rs2281997 T/T genotype and 9 carrying the rs72735260 T/T genotype in the full cohort. Consequently, estimates from recessive and codominant models involving these genotype categories had wider uncertainty and were interpreted accordingly (**Table 2**).

**Table 2:** Genotype distribution, minor allele frequency, and Hardy-Weinberg equilibrium for the four selected variants.

Gene	SNP	Overall genotypes (n=300)	Overall MAF	NVA genotypes (n=184)	NVA MAF	HWE *p* (NVA)	EVA genotypes (n=116)	EVA MAF	HWE *p* (EVA)
ENHO	rs2281997	C/C=226; C/T=63; T/T=11	0.142	C/C=147; C/T=32; T/T=5	0.114	0.065	C/C=79; C/T=31; T/T=6	0.185	0.219
ENHO	rs72735260	G/G=229; G/T=62; T/T=9	0.133	G/G=150; G/T=30; T/T=4	0.103	0.106	G/G=79; G/T=32; T/T=5	0.181	0.527
EDN1	rs5370	G/G=136; G/T=120; T/T=44	0.347	G/G=96; G/T=68; T/T=20	0.293	0.154	G/G=40; G/T=52; T/T=24	0.431	0.349
EDN1	rs2070699	G/G=139; G/T=122; T/T=39	0.333	G/G=91; G/T=76; T/T=17	0.299	0.861	G/G=48; G/T=46; T/T=22	0.388	0.0797

HWE in the NVA group was the primary quality-control assessment; EVA HWE is shown as a sensitivity analysis. The T allele was the minor/risk allele for all four variants.

### 3.5 Individual ENHO and EDN1 variants and early vascular aging

Associations with EVA were tested under five inheritance models, adjusting for age, sex, BMI, and HbA1c. For every SNP, the log-additive model fell within 2 AIC units of the best-fitting model (ΔAIC 0.00–1.14), and log-additive estimates were therefore used for cross-variant comparison, with full model results reported in **Table 3**; **Supplementary Table S3**. For the ENHO variants, rs2281997 was associated with EVA under the dominant model, with carriers of the T allele showing higher adjusted odds than C/C homozygotes (OR 1.857, 95% CI 1.089–3.171; *p* = 0.023; FDR *p* = 0.075). The corresponding log-additive estimate was similar, with an OR of 1.652 per T allele (95% CI 1.067–2.580; *p* = 0.025; FDR *p* = 0.075). A stronger association was observed for rs72735260. In the dominant model, G/T and T/T carriers had about twofold higher odds of EVA than G/G homozygotes (OR 2.076, 95% CI 1.204–3.596; *p* = 0.0087; FDR *p* = 0.030), and the per-T-allele OR was 1.827 (95% CI 1.152–2.934; *p* = 0.011; FDR *p* = 0.030). The association for rs72735260 was significant after FDR correction within the gene, and rs2281997 remained nominally significant (**Figure 2****).** Of the EDN1 variants, rs5370 was most consistently associated across different inheritance models. The adjusted odds of EVA were higher in both G/T heterozygotes (OR 1.863, 95% CI 1.111–3.145) and T/T homozygotes (OR 2.920, 95% CI 1.454–5.942) than in the G/G genotype. The log-additive model demonstrated a dose-dependent increase of EVA risk per T allele (OR 1.740; 95% CI 1.250–2.442; *p* = 0.00115; FDR *p* = 0.0055). The association for rs2070699 was mainly due to the T/T genotype. T/T carriers had higher odds of EVA compared with G/G homozygotes (OR 2.467, 95% CI 1.193-5.187; *p* = 0.015). The recessive comparison also yielded a similar estimate (OR 2.313, 95% CI 1.164-4.672; *p* = 0.017). The log-additive association was more modest (OR 1.443, 95% CI 1.031-2.028; *p* = 0.033; FDR *p* = 0.066), consistent with the lack of a strong effect in heterozygous carriers. Overall, all four variants were associated with EVA in the risk direction, with rs5370 being significant after FDR correction and rs2070699 remaining nominally significant (**Table 3**; **Figure 2**; **Supplementary Table S3**).

**Table 3:** Adjusted associations of individual ENHO and EDN1 variants with early vascular aging under alternative inheritance models.

Gene	SNP	Model	Exact genotype contrast	Adjusted OR (95% CI)	*p* value	FDR *p* value	AIC
ENHO	**rs2281997**	Codominant	C/T vs C/C	1.797 (1.019–3.174)	**0.043**	0.085	402.99
		Codominant	T/T vs C/C	2.234 (0.653–7.971)	0.196	0.235	402.99
		Dominant	C/T + T/T vs C/C	1.857 (1.089–3.171)	**0.023**	0.075	401.10
		Recessive	T/T vs C/C + C/T	1.958 (0.577–6.941)	0.277	0.277	405.08
		Over-dominant	C/T vs C/C + T/T	1.728 (0.983–3.039)	0.057	0.085	402.66
		Log-additive	Per T allele	1.652 (1.067–2.580)	**0.025**	0.075	401.20
ENHO	**rs72735260**	Codominant	G/T vs G/G	2.034 (1.149–3.616)	**0.015**	**0.030**	401.31
		Codominant	T/T vs G/G	2.408 (0.611–10.117)	0.205	0.246	401.31
		Dominant	G/T + T/T vs G/G	2.076 (1.204–3.596)	**0.0087**	**0.030**	399.37
		Recessive	T/T vs G/G + G/T	2.002 (0.512–8.336)	0.314	0.314	405.24
		Over-dominant	G/T vs G/G + T/T	1.952 (1.107–3.455)	**0.021**	**0.031**	400.92
		Log-additive	Per T allele	1.827 (1.152–2.934)	**0.011**	**0.030**	399.71
EDN1	**rs5370**	Codominant	G/T vs G/G	1.863 (1.111–3.145)	**0.019**	**0.025**	397.30
		Codominant	T/T vs G/G	2.920 (1.454–5.942)	**0.0027**	**0.0055**	397.30
		Dominant	G/T + T/T vs G/G	2.104 (1.304–3.431)	**0.0026**	**0.0055**	396.91
		Recessive	T/T vs G/G + G/T	2.147 (1.127–4.134)	**0.021**	**0.025**	400.88
		Over-dominant	G/T vs G/G + T/T	1.397 (0.869–2.247)	0.168	0.168	404.36
		Log-additive	Per T allele	1.740 (1.250–2.442)	**0.0011**	**0.0055**	395.41
EDN1	**rs2070699**	Codominant	G/T vs G/G	1.147 (0.691–1.905)	0.595	0.715	402.26
		Codominant	T/T vs G/G	2.467 (1.193–5.187)	**0.015**	0.052	402.26
		Dominant	G/T + T/T vs G/G	1.383 (0.866–2.219)	0.176	0.263	404.42
		Recessive	T/T vs G/G + G/T	2.313 (1.164–4.672)	**0.017**	0.052	400.54
		Over-dominant	G/T vs G/G + T/T	0.938 (0.582–1.506)	0.790	0.790	406.19
		Log-additive	Per T allele	1.443 (1.031–2.028)	**0.033**	0.066	401.68

Models were adjusted for age, sex, BMI, and HbA1c. FDR p values were calculated separately within each SNP across the six inheritance-model contrasts. AIC, Akaike information criterion; OR, odds ratio; CI, confidence interval. Bold values indicate statistical significance at p < 0.05.

**Figure 2:**
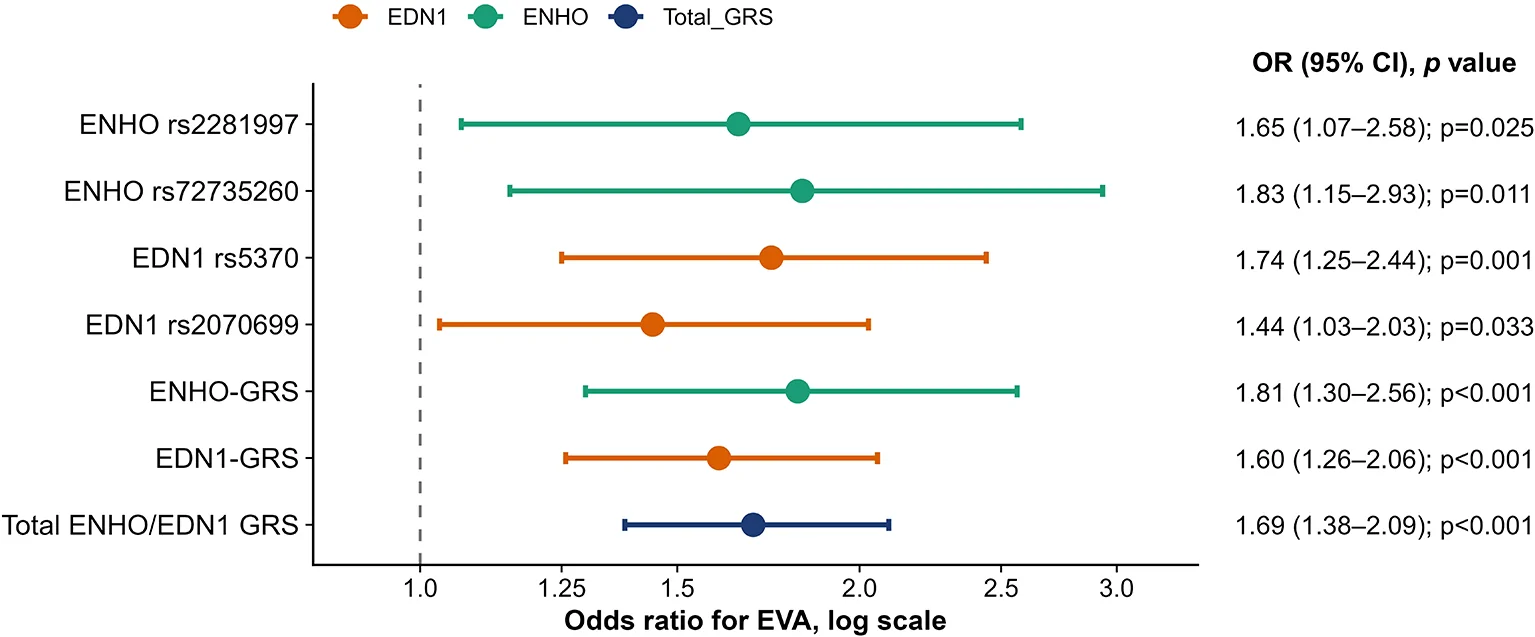
Adjusted associations of individual ENHO and EDN1 variants and genetic risk scores with early vascular aging. Individual SNP estimates are shown under the log-additive model; ENHO-GRS, EDN1-GRS, and Total GRS estimates are per one-unit increase. All models were adjusted for age, sex, BMI, and HbA1c. p value show here is nominal p value.

### 3.6 Individual SNP associations with continuous ePWV

Analysis of continuous ePWV showed positive per-risk-allele coefficients for all four variants, but the strength of evidence differed from that observed for binary EVA (**Supplementary Table S4**). ENHO rs72735260 had the largest log-additive association (β=0.239 m/s per T allele, 95% CI 0.061–0.416; *p* = 0.00869; FDR *p* = 0.0429) and was the only SNP to remain below the 0.05 FDR threshold in this analysis. ENHO rs2281997 was nominally associated with higher ePWV (β=0.181, 95% CI 0.011–0.350; *p* = 0.0368; FDR *p* = 0.0672), with a comparable dominant-model estimate (β=0.235, 95% CI 0.028–0.441; *p* = 0.0262). EDN1 rs2070699 also showed a nominal log-additive association (β=0.162, 95% CI 0.033–0.290; *p* = 0.0137; FDR *p* = 0.0537), whereas EDN1 rs5370 was directionally positive but not statistically significant (β=0.099, 95% CI −0.027 to 0.225; *p* = 0.122; FDR *p* = 0.244). These continuous-outcome estimates are reported in full across inheritance models in **Supplementary Table S4**.

### 3.7 Linkage disequilibrium and haplotype associations

Pairwise linkage disequilibrium was weak for the ENHO pair and absent for the EDN1 pair. ENHO rs2281997–rs72735260 had D′=0.251 and r²=0.0016 (p=0.326), whereas EDN1 rs5370–rs2070699 had D′=0.0077 and r²<0.001 (p=0.922). These findings indicate that the allele dosages of the two variants within each gene were only weakly correlated in the present cohort. The ENHO global haplotype test was borderline (p=0.060). The common C–G haplotype had a frequency of 0.7391 and showed a protective-direction signal (p=0.0097), whereas the T–G and C–T haplotypes showed weaker trends (p=0.079 and p=0.089, respectively); the rare T–T haplotype had no association (frequency 0.0141; p=0.880). Because the global ENHO haplotype test did not reach significance, haplotype-specific estimates for this gene are reported as exploratory. The EDN1 global haplotype test was significant (p=0.025). The T–G haplotype, with a frequency of 0.2320, carried the principal risk-direction signal (p=0.0043), while the G–G haplotype showed a protective trend (frequency 0.4347; p=0.052). Complete frequencies and pair-specific results are provided in **Supplementary Table S5** and visualized in **Supplementary Figure S2**.

### 3.8 Genetic risk scores and vascular-aging outcomes

Of the 300 participants, 120 (40.0%) were classified as having low genetic burden, 86 (28.7%) as having intermediate burden, and 94 (31.3%) as having high burden. EVA prevalence increased across these categories from 25.0% in the low-burden group to 44.2% in the intermediate-burden group and 51.1% in the high-burden group. The Total GRS was consistently associated with EVA across all sequential models (**Table 4**; **Figure 2**). The OR per additional risk allele was 1.668 in the unadjusted model, 1.674 after adjustment for age, sex, and BMI, and 1.691 after further adjustment for HbA1c. In the fully adjusted model, each one-unit increase in Total GRS corresponded to a 69.1% increase in the odds of EVA (OR 1.691, 95% CI 1.381–2.094; *p* < 0.001).

**Table 4:** Association of ENHO-GRS, EDN1-GRS, and Total GRS with early vascular aging and continuous ePWV.

Comparison	Exposure	Outcome	Effect (95% CI)	p
Total GRS	Total GRS (per unit)	EVA	OR 1.691 (1.381–2.094)	**<0.001**
GRS category	Intermediate vs low		OR 2.364 (1.309–4.314)	**0.004**
GRS category	High vs low		OR 3.473 (1.945–6.309)	**<0.001**
Gene-specific GRS	ENHO-GRS (per unit)		OR 1.814 (1.298–2.564)	**<0.001**
Gene-specific GRS	EDN1-GRS (per unit)		OR 1.602 (1.258–2.057)	**<0.001**
Total GRS	Total GRS (per unit)	ePWV	β 0.153 (0.083–0.223)	**<0.001**
Gene-specific GRS	ENHO-GRS (per unit)		β 0.220 (0.096–0.345)	**<0.001**
Gene-specific GRS	EDN1-GRS (per unit)		β 0.129 (0.040–0.218)	**0.005**

Logistic regression effects are presented as odds ratios, and linear regression effects as β coefficients. Models were adjusted for age, sex, BMI, and HbA1c. Bold values indicate statistical significance at p < 0.05.

A dose–response pattern was also evident across Total GRS categories. Compared with participants in the low-GRS group, those in the intermediate-GRS group had more than twice the odds of EVA (OR 2.364, 95% CI 1.309–4.314; *p* = 0.00459), while those in the high-GRS group had more than threefold higher odds (OR 3.473, 95% CI 1.945–6.309; *p* < 0.001). Both pathway-specific scores contributed to this association: each one-unit increase in ENHO-GRS was associated with higher odds of EVA (OR 1.814, 95% CI 1.298–2.564; *p* < 0.001), as was each one-unit increase in EDN1-GRS (OR 1.602, 95% CI 1.258–2.057; *p* < 0.001) (**Table 4**; **Figure 2**).

The genetic-burden association was also observed when vascular aging was examined as a continuous phenotype. Total GRS was associated with higher ePWV in the crude model (β=0.156, 95% CI 0.086–0.225; *p* < 0.001) and after adjustment for age, sex, BMI, and HbA1c (β=0.153, 95% CI 0.083–0.223; p < 0.001). The ENHO-GRS and EDN1-GRS were likewise associated with higher ePWV, with β coefficients of 0.220 and 0.129, respectively (**Table 4**).

### 3.9 Genetic burden, adropin, ET-1, and the adropin–endothelin-1 imbalance

The gene-specific scores were associated with their corresponding peptide axes (**Table 5**; **Figure 3**). Each one-unit increase in ENHO-GRS was associated with lower ln(adropin) (β=−0.592, 95% CI −0.743 to −0.442; *p* < 0.001), whereas each one-unit increase in EDN1-GRS was associated with higher ln(ET-1) (β=0.299, 95% CI 0.186–0.411; *p* < 0.001). Total GRS was associated with lower ln(adropin) levels (β=−0.287; *p* < 0.001) and higher ln(ET-1) levels (β=0.268; *p* < 0.001), resulting in a higher adropin–endothelin-1 imbalance index (AEI; β=0.541, 95% CI 0.393–0.690; *p* < 0.001). The category-based distributions in **Figure 3A** show progressively lower adropin across ENHO-GRS groups, higher ET-1 across EDN1-GRS groups, and higher AEI across Total GRS groups (**Figure 3**).

**Table 5:** Association of SNP and GRS burden with adropin, endothelin-1, and AEI.

Exposure	Outcome	β (95% CI)	*p* value	FDR *p* value
ENHO-GRS	ln(adropin)	−0.592 (−0.743, −0.442)	**<0.001**	–
EDN1-GRS	ln(ET-1)	0.299 (0.186, 0.411)	**<0.001**	–
Total GRS	AEI	0.541 (0.393, 0.690)	**<0.001**	–
Total GRS	ln(adropin)	−0.287 (−0.374, −0.199)	**<0.001**	–
Total GRS	ln(ET-1)	0.268 (0.179, 0.357)	**<0.001**	–
rs2281997 (T dosage)	ln(adropin)	−0.739 (−0.943, −0.535)	**<0.001**	**<0.001**
rs72735260 (T dosage)	ln(adropin)	−0.359 (−0.589, −0.130)	**0.002**	**0.004**
rs2070699 (T dosage)	ln(ET-1)	0.330 (0.165, 0.495)	**<0.001**	**<0.001**
rs5370 (T dosage)	ln(ET-1)	0.272 (0.110, 0.434)	**0.001**	**0.002**
rs2281997 (T dosage)	AEI	0.997 (0.635, 1.360)	**<0.001**	**<0.001**
rs2070699 (T dosage)	AEI	0.534 (0.251, 0.816)	**<0.001**	**<0.001**

β values are linear-regression coefficients from models adjusted for age, sex, BMI, and HbA1c. Adropin and ET-1 were natural-log transformed. Bold values indicate statistical significance at p < 0.05.

**Figure 3:**
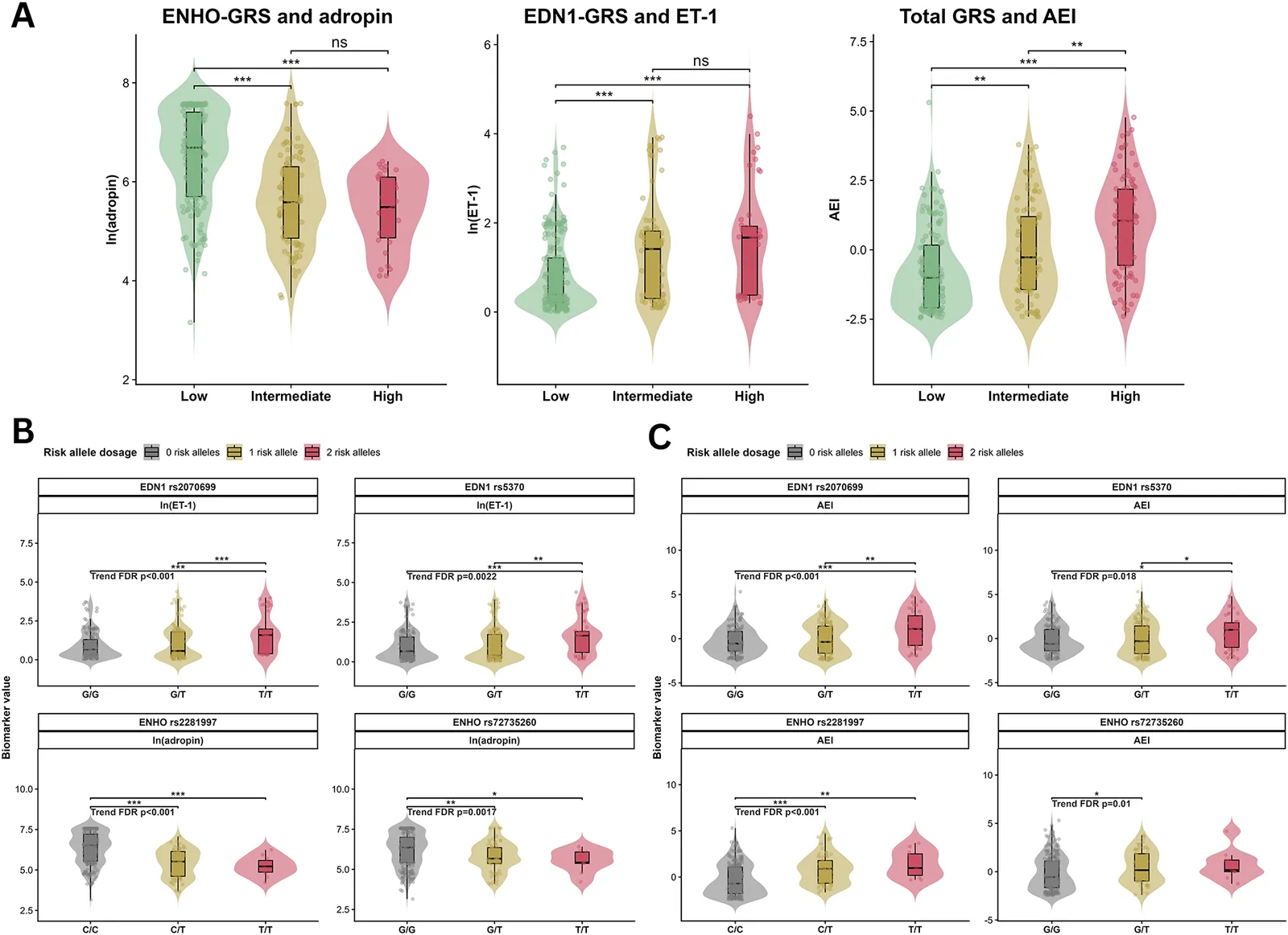
Genetic burden across the ENHO–EDN1 axis is associated with encoded peptide concentrations and adropin–endothelin-1 imbalance. **(A)** Distributions of ln(adropin) across low, intermediate, and high *ENHO*-GRS categories; ln(ET-1) across *EDN1*-GRS categories; and the adropin–endothelin-1 imbalance index (AEI) across Total GRS categories. **(B)** Pathway-specific genotype–biomarker associations showing ln(ET-1) across *EDN1* rs2070699 and rs5370 genotypes and ln(adropin) across *ENHO* rs2281997 and rs72735260 genotypes. **(C)** AEI distributions across genotypes of the four selected variants. Significance is indicated as *p < 0.05, **p < 0.01, and ***p < 0.001; ns, not significant.

At the individual-variant level, both ENHO risk alleles were associated with lower ln(adropin), and both EDN1 risk alleles were associated with higher ln(ET-1). Cross-axis associations were also present for rs2281997 with higher ln(ET-1) (β=0.283; FDR p=0.016) and rs2070699 with lower ln(adropin) (β=−0.217; FDR p=0.014), whereas rs72735260–ET-1 and rs5370–adropin estimates were not significant. All four SNPs were positively associated with AEI, and the largest AEI shift was observed for rs2281997, followed by rs2070699. Genotype-stratified peptide and AEI distributions, together with trend estimates, are provided in **Supplementary Table S6**; **Figures 3B, C**.

### 3.10 Distributional flow from Total GRS through AEI to vascular-aging status

Participant-level flows across Total GRS categories, AEI tertiles, and vascular-aging status are shown in **Figure 4A**. Low genetic burden was predominantly associated with low or intermediate AEI and more frequently culminated in NVA. In contrast, high genetic burden was accompanied by a shift toward the high-AEI tertile and a greater proportion of EVA. The highest proportion of EVA was observed in the combined high-GRS/high-AEI pathway, and NVA dominated the low-GRS/low-AEI pathway (**Figure 4**).

**Figure 4:**
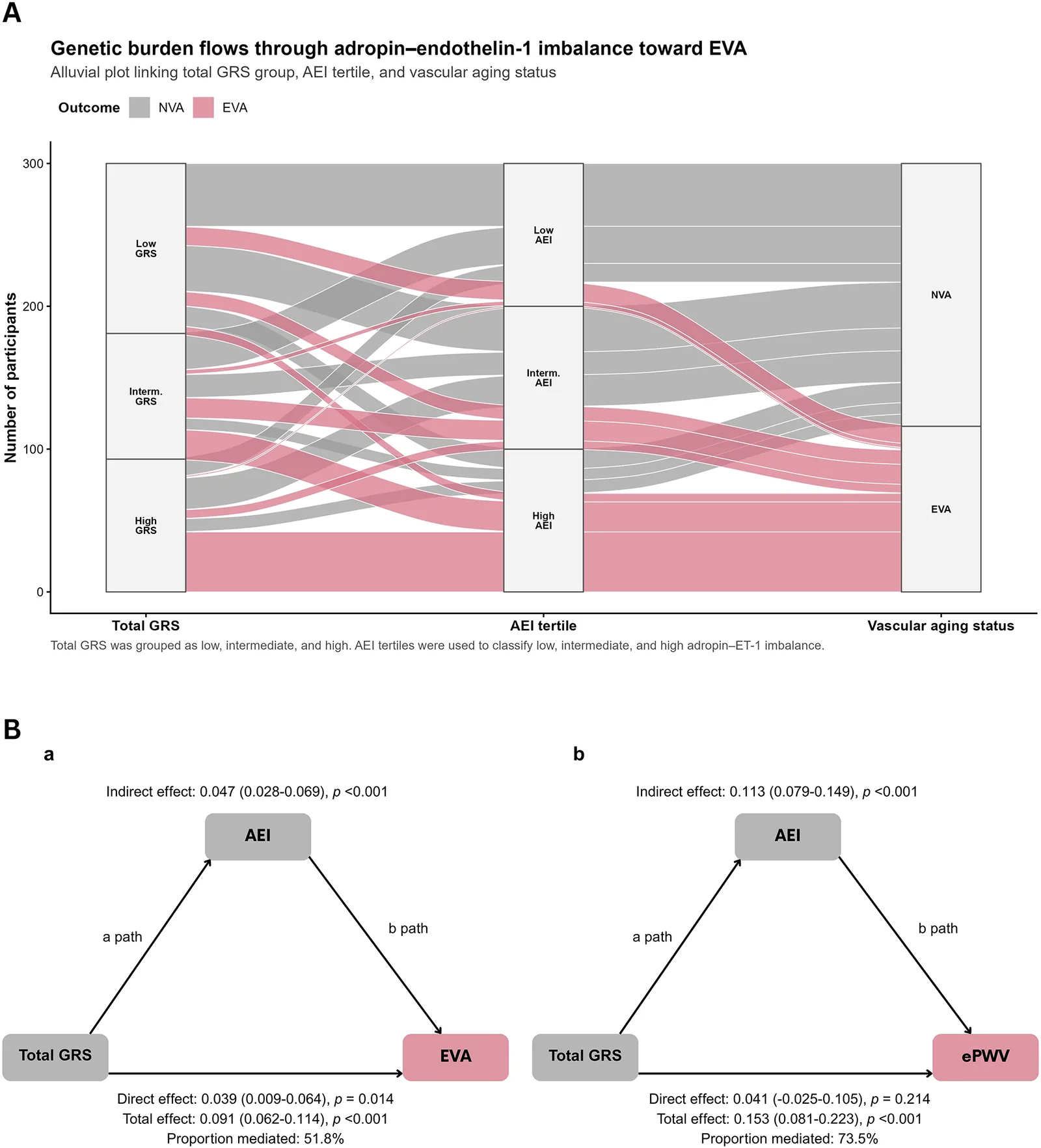
Genetic burden flows through adropin–endothelin-1 imbalance toward early vascular aging. **(A)** Alluvial plot showing the distribution of participants from Total genetic risk score (GRS) category to adropin–endothelin-1 imbalance index (AEI) tertile and vascular-aging status. **(B)** Mediation diagrams for Total GRS → AEI → EVA and Total GRS → AEI → ePWV.

### 3.11 Statistical mediation by the adropin–endothelin-1 imbalance

In the primary bootstrap mediation model adjusted for age, sex, BMI, and HbA1c, AEI significantly mediated the association between Total GRS and EVA (**Figure 4B**; **Supplementary Table S7**). The estimated indirect effect through AEI was 0.047 (95% CI 0.028–0.069; *p* < 0.001), while the residual direct effect was 0.039 (95% CI 0.009–0.064; *p* = 0.0144). The total effect was 0.091 (95% CI 0.062–0.114; *p* < 0.001), of which 51.8% was statistically mediated through AEI (95% CI 30.5%–86.8%; *p* < 0.001). These findings indicate that approximately half of the observed association between Total GRS and EVA was accounted for by the adropin–endothelin-1 imbalance, with a smaller but significant direct association remaining. When ePWV was analyzed as a continuous outcome, the indirect effect through AEI remained statistically significant (ACME 0.113, 95% CI 0.079–0.149; p<0.001), whereas the direct effect was not significant (ADE 0.041, 95% CI −0.025 to 0.105; p=0.214). The total effect was 0.153 (95% CI 0.081–0.223; p<0.001), with an estimated proportion mediated of 73.5%. However, the confidence interval for the proportion mediated extended beyond the theoretical range of 0 to 1 (0.490–1.283), indicating considerable imprecision; this estimate was therefore interpreted cautiously (**Figure 4B**; **Supplementary Table S7**). Gene-specific models also showed significant indirect effects. ENHO-GRS was indirectly associated with both EVA and ePWV through ln(adropin), while the corresponding direct effects were not significant. EDN1-GRS was indirectly associated with both outcomes through ln(ET-1), and a residual direct effect remained for EVA but not for ePWV. Full effect estimates, confidence intervals, proportions mediated, and sensitivity analysis are provided in **Supplementary Table S7**. Given the cross-sectional design, these findings are interpreted as statistical rather than causal mediation.

### 3.12 Exploratory discrimination of early vascular aging

The clinical model showed limited discrimination, with an AUC of 0.590 (95% CI 0.523 to 0.657). The Total GRS alone had an AUC of 0.661 (95% CI 0.600–0.722), and the addition of it to the clinical model increased the AUC to 0.686 (95% CI 0.624–0.748; DeLong p=0.010). AEI alone had a higher AUC of 0.776 (95% CI 0.720–0.833). The clinical + AEI model retained an AUC of 0.776 and significantly outperformed the clinical model alone (DeLong *p* < 0.001). At the optimal Youden threshold of 0.382, AEI achieved a sensitivity of 74.1% and a specificity of 76.1%. The full clinical + Total GRS + AEI model produced the highest AUC of 0.779 (95% CI 0.721–0.836; DeLong *p* < 0.001 vs the clinical model), with a sensitivity of 73.3% and a specificity of 74.5%. However, its discrimination did not differ significantly from that of the clinical + AEI model, indicating that AEI accounted for most of the discriminatory performance of the integrated model. In contrast, Total GRS provided a clearer incremental improvement when added to the clinical model alone (**Figure 5**; **Supplementary Table S8**).

**Figure 5:**
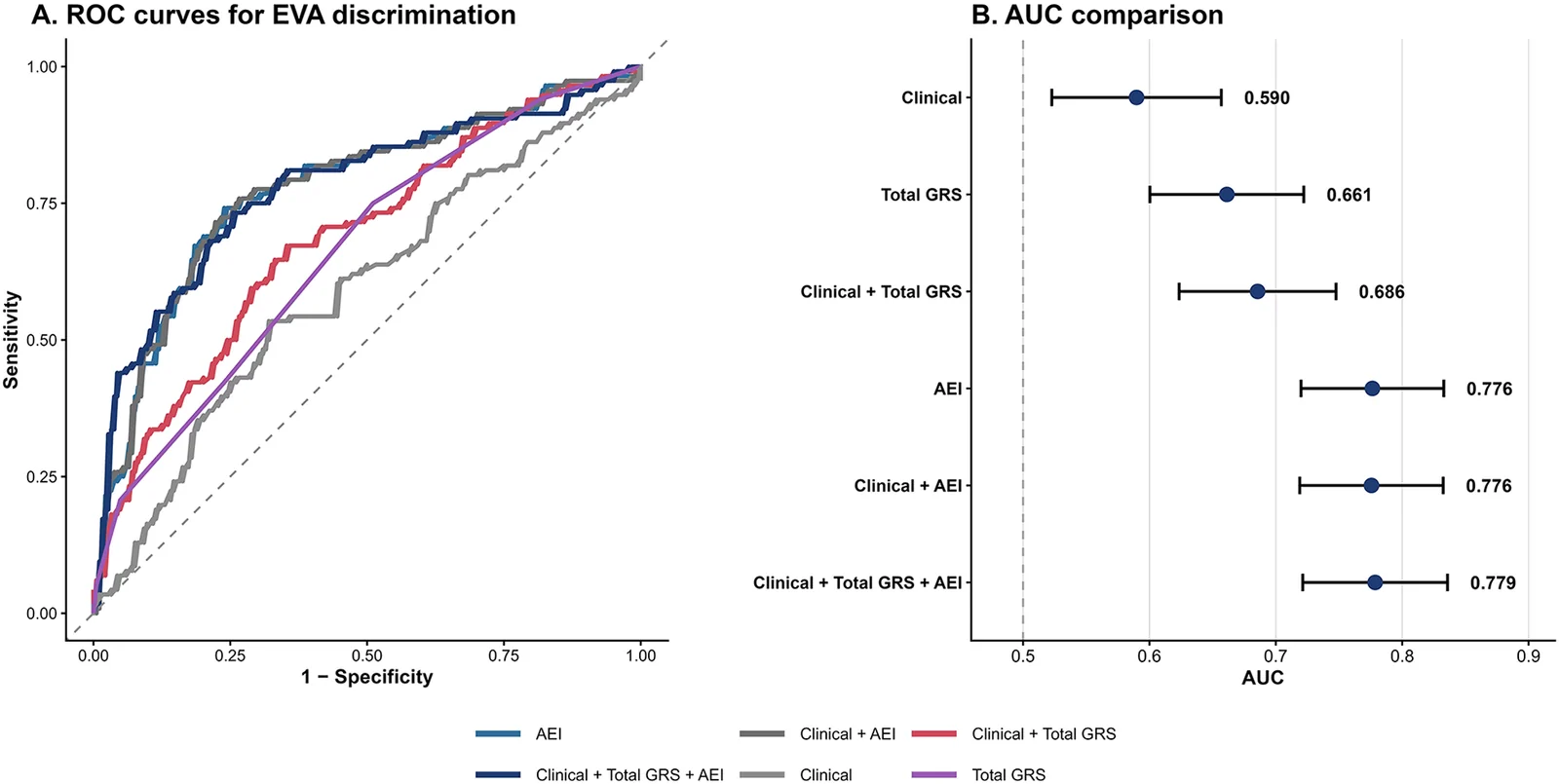
Exploratory discrimination of early vascular aging using clinical, genetic, biomarker, and integrated models. **(A)** shows ROC curves; **(B)** compares AUC estimates and 95% confidence intervals. Clinical model included age, sex, and BMI.

### 3.13 In silico annotation and cohort-level concordance

Functional annotation was interpreted alongside the observed genotype–biomarker and genotype–vascular-aging associations (**Table 6**; **Supplementary Figure S3**). ENHO rs2281997 is an intronic variant assigned RegulomeDB rank 1f and located within a splice-associated polypyrimidine-tract region, although its SpliceAI scores were low. Increasing T-allele dosage was associated with lower adropin levels, higher AEI, greater odds of EVA, and higher ePWV. ENHO rs72735260 is a 3′-UTR variant with RegulomeDB rank 1f and SpliceAI scores of 0.00. Its T allele was associated with lower adropin levels, higher odds of EVA, and increased ePWV, while the association with AEI was comparatively weaker. EDN1 rs5370 is a missense variant resulting in the Lys198Asn substitution, is reported in ClinVar, and has a RegulomeDB rank of 1f. Increasing T-allele dosage was associated with higher ET-1 levels, a higher AEI, and greater odds of EVA, whereas its association with continuous ePWV was positive but not statistically significant. EDN1 rs2070699 is an intronic variant reported in ClinVar and assigned RegulomeDB rank 1b, representing the strongest regulatory evidence among the four variants. Its T allele was associated with higher ET-1, higher AEI, increased odds of EVA, and higher ePWV. The integrated annotation and cohort-concordance matrix is presented in **Supplementary Figure S3**. As these annotations were performed after variant selection, they support biological plausibility but do not establish functional causality.

**Table 6:** In silico annotation and observed genotype–biomarker/vascular-aging concordance of the selected ENHO and EDN1 variants.

Gene	SNP	Position (GRCh38)	Risk allele	Variant context	Database and in silico annotation	Observed cohort concordance	Interpretation
ENHO	rs2281997	chr9:34521869	T	ENHO intron variant	Not reported in ClinVar; population frequency: T frequency approx. 0.28-0.29 in large population datasets; RegulomeDB: Rank 1f; score 0.19549; SpliceAI: Splice polypyrimidine tract variant; low SpliceAI scores: acceptor loss 0.01, acceptor gain 0.03, donor gain 0.03.	• T-risk dosage associated with lower adropin and higher AEI. • T-risk dosage associated with higher EVA odds and higher ePWV.	Concordant with a possible ENHO transcript-level or regulatory effect; splice prediction was low but the variant lies in a splice-relevant intronic context
ENHO	rs72735260	chr9:34521190	T	ENHO 3’UTR variant; DNAI1–500 bp downstream variant	Not reported in ClinVar; population frequency: T frequency approx. 0.07-0.10 in large population datasets; RegulomeDB: Rank 1f; score 0.22271; SpliceAI: 3’UTR variant.	• T-risk dosage associated with lower adropin; AEI association weaker at genotype level. • T-risk dosage associated with higher EVA odds and higher ePWV.	Concordant with a possible ENHO 3’UTR regulatory effect rather than splice alteration
EDN1	rs5370	chr6:12296022	T	EDN1 missense variant	ClinVar: Reported in ClinVar; population frequency: T frequency approx. 0.21-0.23 in large population datasets; RegulomeDB: Rank 1f; score 0.55436.	• T-risk dosage associated with higher ET-1 and higher AEI trend. • T-risk dosage associated with higher EVA odds; continuous ePWV direction positive but nonsignificant.	Biologically plausible EDN1 coding variant with cohort-level concordance for ET-1 and EVA
EDN1	rs2070699	chr6:12292539	T	EDN1 intron variant	ClinVar: Reported in ClinVar; population frequency: T frequency approx. 0.35-0.46 in large population datasets; RegulomeDB: Rank 1b; score 0.53508; SpliceAI: EDN1 intron variant.	• T-risk dosage associated with higher ET-1 and higher AEI. • T-risk dosage associated with higher EVA odds and higher ePWV.	Concordant with a regulatory/intronic EDN1 effect; strongest RegulomeDB rank among the four variants

## 4 Discussion

In this cohort of adults with and without T2DM, this study investigated whether common variation in ENHO and EDN1 is associated with the corresponding circulating peptides and vascular aging. Higher ENHO genetic burden was associated with lower circulating adropin, whereas higher EDN1 genetic burden was associated with higher circulating ET-1. The combined genetic risk score was also associated with greater adropin to ET-1 imbalance and a less favorable vascular-aging phenotype. These associations were broadly maintained when vascular aging was assessed continuously using ePWV, although the effects of individual variants were not fully consistent between the binary and continuous outcome models. The results therefore point more strongly to a pathway-level relationship than to a large effect from any single variant. This pathway-level pattern agrees with the known vascular effects of adropin and ET-1. Adropin supports endothelial nitric oxide production through eNOS activation, while circulating and arterial adropin concentrations decline with advancing age (47). ET-1 expression and ET-1-mediated vasoconstrictor tone increase with age and contribute to endothelial dysfunction and hypertension (48). A genetic profile associated with lower adropin and greater ET-1 activity would therefore be expected to accompany a less favorable vascular phenotype. Previous studies have generally examined ENHO and EDN1 in relation to individual biomarkers or cardiometabolic outcomes, while current EVA prediction models remain largely based on clinical variables.

The ENHO results can be interpreted in the context of the limited genetic evidence available for these variants. Grzegorzewska et al. reported that the rs2281997 T allele was associated with a hyper-LDL-cholesterolemic profile among patients undergoing hemodialysis, while major-allele homozygotes tended to have higher circulating adropin concentrations than minor-allele carriers (31). This pattern agrees with the present finding that increasing rs2281997 T-allele dosage was associated with lower adropin. The direction of the association has not been uniform across clinical settings. The same allele was associated with less atherogenic dyslipidemia and lower cardiovascular mortality in a selected hemodialysis subgroup (31). Evidence for rs72735260 is more limited because it has mainly been evaluated together with rs2281997, with little evidence regarding its independent phenotypic effect. Clinical studies of circulating adropin provide additional support for the direction of the ENHO associations. Lower adropin concentrations have been reported in patients with angiographic coronary atherosclerosis, both with and without diabetes, stable coronary artery disease, and coronary slow-flow phenomenon, a marker of coronary microvascular dysfunction (49–51). Reduced adropin has also been observed in primary hypertension and hypertensive target-organ damage (52, 53). These studies link lower adropin with several adverse vascular phenotypes and support the biological interpretation of the present findings. Genetic variation, however, is likely to explain only part of the variation in circulating adropin between individuals.

The literature on EDN1 rs5370 is extensive but less consistent. Previous studies have associated rs5370 with blood pressure among individuals with overweight, large-artery and ischemic stroke, and coronary artery disease (32–34). However, population-based studies from China and Myanmar did not identify significant associations with hypertension or related cardiometabolic outcomes (54, 55). Experimental findings also do not support a simple direct effect of the Lys198Asn substitution on ET-1 secretion. Tanaka et al. observed no genotype-dependent differences in ET-1 or big ET-1 secretion in transfected cells or in circulating ET-1 concentrations among individuals with hypertension (56). More recent functional studies have identified other regulatory variants within EDN1 that alter transcriptional activity (57). This raises the possibility that rs5370 reflects an underlying regulatory haplotype rather than directly changing ET-1 secretion through the amino acid substitution. The increased EVA risk observed in the present study agrees with several previously reported positive associations (32–34, 58). The absence of an association in other populations suggests that the effect is modest and may depend on genetic background, environmental exposure, or the phenotype being studied. The evidence for EDN1 rs2070699 is more limited and also varies across populations. Among neonates with persistent pulmonary hypertension, the T allele was associated with higher circulating ET-1 concentrations and greater disease susceptibility, providing partial support for the T-allele risk orientation reported in the present study (35). Conversely, the G allele has been associated with ischemic stroke among Chinese men (59), and other studies have described associations with pulmonary, renal, and inflammatory phenotypes. The rs2070699 association observed in this cohort should therefore be interpreted in relation to the studied population and vascular-aging phenotype rather than as a universal allele effect.

At the individual-variant level, the strongest multiple-testing-adjusted evidence was observed for rs5370 and rs72735260, whereas the associations involving rs2281997 and rs2070699 were mainly nominal. These differences at the variant level support the use of gene-specific and combined genetic scores to represent the accumulated burden across the two pathways. Minimal linkage disequilibrium within each gene pair indicated that the variants contributed largely nonredundant information. An unweighted scoring method was selected because validated South Asian effect estimates were unavailable. Deriving weights from the same cohort would also have increased the risk of overfitting. A similar score-based approach has previously shown an independent association between cardiometabolic genetic burden and carotid-femoral pulse-wave velocity (60).

The score-based analysis produced the clearest agreement between genetic burden and the encoded circulating peptides. Higher ENHO-GRS was associated with lower adropin, higher EDN1-GRS was associated with higher ET-1, and Total GRS was associated with a shift toward ET-1 predominance relative to adropin. Agreement across these levels strengthens the biological interpretation through epidemiologic triangulation, although it does not establish causality (61). The opposite vascular actions of adropin and ET-1 also provide a biological basis for combining them as an imbalance measure and help explain the association between Total GRS and AEI. Several variants also showed cross-axis associations. ENHO variants were associated with ET-1, while EDN1 variants were associated with adropin. These relationships may arise from shared regulation through nitric oxide bioavailability, metabolic influences, or endothelial feedback because both peptides interact with the eNOS and nitric oxide pathway (14, 47, 48). Shared endothelial signaling could therefore produce some degree of interaction between the two peptide systems. The cross-axis findings were not consistent across all variants and were secondary to the prespecified gene-to-encoded-peptide analysis. They should be treated as exploratory until they are replicated independently.

The mediation results followed the same gene, peptide, and vascular phenotype sequence. AEI statistically accounted for part of the association between Total GRS and both binary EVA and continuous ePWV. Cross-sectional mediation analysis cannot establish whether changes in peptide imbalance preceded the vascular phenotype. Estimates from cross-sectional data may also differ from the underlying longitudinal process even when the specified model is statistically appropriate (62). The proportion mediated is a ratio measure and may become unstable when the total or direct effect is small (63). The indirect effect and its bootstrap confidence interval therefore provide a more reliable basis for interpretation than the proportion mediated alone. The results are statistically compatible with the proposed pathway from genetic burden through peptide imbalance to vascular aging, but they do not demonstrate causal mediation. Prospective and mechanistic studies showing that adropin and ET-1 influence vascular function support the proposed biological direction (14, 15, 64). Confirmation would require longitudinal studies with repeated peptide and vascular measurements and analysis addressing unmeasured mediator-to-outcome confounding. The ROC v provide a practical extension of these findings. AEI showed greater discrimination for EVA than Total GRS, while adding Total GRS to the AEI-based model produced little further improvement. This finding agrees with cardiovascular prediction studies in which genetic risk scores have generally produced only modest improvements beyond established clinical or biochemical predictors (65, 66). The strength of this study was the availability of genetic, peptide, and vascular measurements in the same participants. The prespecified analytical sequence progressed from individual variants to gene-specific scores, Total GRS, encoded peptides, AEI, and vascular phenotype. This structure allowed each result to be assessed within the same biological pathway rather than as an isolated statistical association.

The findings must also be interpreted within the limits of the study design. The modest sample size, particularly in the minor-homozygote and recessive-genotype groups, increased statistical uncertainty and may have inflated some effect estimates. Recruitment from a single tertiary center in South India limits generalizability to other populations and clinical settings. Arterial stiffness was estimated using ePWV rather than measured directly using carotid-femoral pulse-wave velocity. Although ePWV is practical and has prognostic value, it is derived partly from age and mean arterial pressure and is not interchangeable with directly measured carotid-femoral pulse-wave velocity. The cross-sectional design also prevents confirmation of the temporal sequence between peptide imbalance and vascular aging. In addition, the ROC analysis lacked external validation; therefore, their discriminatory performance may be optimistic and requires confirmation in an independent cohort. The four-SNP candidate panel represents only a small part of the genetic variation within ENHO, EDN1, and the wider pathways regulating endothelial function. Larger multicenter studies in independent and ancestrally diverse populations are needed to determine the reproducibility and magnitude of these associations.

## 5 Conclusion

Common variation in ENHO and EDN1 was associated with vascular aging phenotype in our cohort. These associations were accompanied by directionally consistent differences in circulating adropin, ET-1, and their relative imbalance. The agreement between genetic burden, the corresponding peptide concentrations, and vascular phenotype provides the most consistent support in this dataset for the proposed adropin–endothelin-1 pathway. This pattern agrees with physiological and clinical evidence describing adropin as vasoprotective and ET-1 as a contributor to vascular dysfunction. Individual variant effects were modest and were not fully consistent across the binary and continuous vascular outcomes. The adropin and endothelin-1 axis may represent an inherited and biochemical correlate of premature vascular aging. Independent replication is required before the individual variants, genetic risk scores, or AEI can be considered for clinical application.

## Data Availability

The original contributions presented in the study are included in the article/**Supplementary Material**. Further inquiries can be directed to the corresponding authors.

## References

[1] Roman G Pantea Stoian A . Cardiovascular risk/disease in type 2 diabetes mellitus. In: Type 2 Diabetes - From Pathophysiology to Cyber Systems. London: IntechOpen (2021). doi: 10.5772/intechopen.97422

[2] Jeon JY Kim DJ . Cardiovascular disease in patients with type 2 diabetes. J Diabetes Investig. (2022) 13:614–6. doi: 10.1111/jdi.13742 34953095 PMC9017622

[3] Hill MA Yang Y Zhang L Sun Z Jia G Parrish AR . Insulin resistance, cardiovascular stiffening and cardiovascular disease. Metab Clin Exp. (2021) 119:154766. doi: 10.1016/j.metabol.2021.154766 33766485

[4] Pascual-Morena C Cavero-Redondo I Martínez-García I Rodríguez-Gutiérrez E Lucerón-Lucas-Torres M Moreno-Herráiz N . Exploring the influence of insulin resistance on arterial stiffness in healthy adults: From the metabolic and cardiovascular health insights of the EVasCu study. Nutrients. (2024) 16(6):791. doi: 10.3390/nu16060791 38542702 PMC10974428

[5] Craciun LM Buleu F Iurciuc S Popa DI Pop GN Goanta F . Arterial stiffness and early cardiac dysfunction in type 2 diabetes mellitus: A potential role for 25 OH vitamin D3 deficiency. Medicina (Kaunas). (2025) 61(8):1349. doi: 10.3390/medicina61081349 40870394 PMC12387657

[6] Staef M Ott C Kannenkeril D Striepe K Schiffer M Schmieder RE . Determinants of arterial stiffness in patients with type 2 diabetes mellitus: A cross sectional analysis. Sci Rep. (2023) 13(1):8944. doi: 10.1038/s41598-023-35589-4 37268640 PMC10238522

[7] Cunha P Boutouyrie P Nilsson P Laurent S . Early vascular ageing (EVA): Definitions and clinical applicability. Curr Hypertension Rev. (2017) 13:8–15. doi: 10.2174/1573402113666170413094319 28412914

[8] Bruno RM Nilsson PM Engström G Wadström BN Empana JP Boutouyrie P . Early and supernormal vascular aging. Hypertension. (2020) 76:1616–24. doi: 10.1161/hypertensionaha.120.14971 32895017

[9] Greve SV Blicher MK Kruger R Sehestedt T Gram-Kampmann E Rasmussen S . Estimated carotid–femoral pulse wave velocity has similar predictive value as measured carotid–femoral pulse wave velocity. J Hypertens. (2016) 34:1279–89. doi: 10.1097/hjh.0000000000000935 27088638

[10] Cheng W Kong F Pan H Luan S Yang S Chen S . Superior predictive value of estimated pulse wave velocity for all-cause and cardiovascular disease mortality risk in U.S. general adults. BMC Public Health. (2024) 24(1):600. doi: 10.1186/s12889-024-18071-2 38402165 PMC10893621

[11] Alves-Cabratosa L Elosua-Bayés M Martí-Lluch R Blanch J Tornabell-Noguera È Garcia-Gil M . Cardiovascular risk age reflects arterial status: Middle-aged people showed equivalent arterial stiffness to older people in the same risk category. J Atheroscl Thromb. (2023) 31:626–40. doi: 10.5551/jat.64541 38171907 PMC11079498

[12] Ali II D'souza C Singh J Adeghate E . Adropin’s role in energy homeostasis and metabolic disorders. Int J Mol Sci. (2022) 23(15):8318. doi: 10.3390/ijms23158318 35955453 PMC9369016

[13] Lovren F Pan Y Quan A Singh KK Shukla PC Gupta M . Adropin is a novel regulator of endothelial function. Circulation. (2010) 122:S185–92. doi: 10.1161/circulationaha.109.931782 20837912

[14] Yang C Lavayen BP Liu L Sanz BD DeMars KM Larochelle J . Neurovascular protection by adropin in experimental ischemic stroke through an endothelial nitric oxide synthase-dependent mechanism. Redox Biol. (2021) 48:102197. doi: 10.1016/j.redox.2021.102197 34826783 PMC8633041

[15] Kwon O Andtbacka R Hyngstrom J Richardson R . Vasodilatory function in human skeletal muscle feed arteries with advancing age: The role of adropin. J Physiol. (2019) 597(7):1791–804. doi: 10.1113/jp277410 30690728 PMC6441888

[16] Dodd WS Patel D Laurent D Lucke-Wold B Hosaka K Johnson RD . Subarachnoid hemorrhage-associated brain injury and neurobehavioral deficits are reversed with synthetic adropin treatment through sustained Ser1179 phosphorylation of endothelial nitric oxide synthase. Front Stroke. (2024) 3. doi: 10.3389/fstro.2024.1371140 39345725 PMC11434178

[17] Houde M Desbiens L D'Orléans-Juste P . Endothelin-1: Biosynthesis, signaling and vasoreactivity. Adv Pharmacol. (2016) 77:143–75. doi: 10.1016/bs.apha.2016.05.002 27451097

[18] Moedt E Wasehuus VS Heerspink H . Selective endothelin A receptor antagonism in chronic kidney disease: Improving clinical application. Nephrol Dialysis Transplant. (2025) 40:i37–46. doi: 10.1093/ndt/gfae214 39907539 PMC11795649

[19] Marasciulo FL Montagnani M Potenza M . Endothelin-1: The yin and yang on vascular function. Curr Med Chem. (2006) 13:1655–65. doi: 10.2174/092986706777441968 16787211

[20] Fujie S Hasegawa N Sato K Fujita S Sanada K Hamaoka T . Aerobic exercise training-induced changes in serum adropin level are associated with reduced arterial stiffness in middle-aged and older adults. Am J Physiol Heart Circ Physiol. (2015) 309:H1642–7. doi: 10.1152/ajpheart.00338.2015 26371163

[21] Lin S Su I Lee C Lai Y Hsu B . The correlation between serum endothelin-1 levels and aortic stiffness in patients with non-dialysis chronic kidney disease. Renal Failure. (2026) 48(1):2641961. doi: 10.1080/0886022x.2026.2641961 41820016 PMC12983806

[22] Farrah TE Melville V Czopek A Fok H Bruce L Mills NL . Arterial stiffness, endothelial dysfunction and impaired fibrinolysis are pathogenic mechanisms contributing to cardiovascular risk in ANCA-associated vasculitis. Kidney Int. (2022) 102(5):1115–26. doi: 10.1016/j.kint.2022.07.026 35998848

[23] Gu X Li H Zhu X Gu H Chen J Wang L . Inverse correlation between plasma adropin and ET-1 levels in essential hypertension: A cross-sectional study. Med (Baltimore). (2015) 94:e1712. doi: 10.1097/MD.0000000000001712 26448026 PMC4616732

[24] Fan Z Zhang Y Zou F Xu T Pan P Hu C . Serum adropin level is associated with endothelial dysfunction in patients with obstructive sleep apnea and hypopnea syndrome. Sleep Breathing. (2020) 25:117–23. doi: 10.1007/s11325-020-02072-7 32253609

[25] Notsu Y Yano S Shibata H Nagai A Nabika T . Plasma arginine/ADMA ratio as a sensitive risk marker for atherosclerosis: Shimane CoHRE study. Atherosclerosis. (2015) 239:61–6. doi: 10.1016/j.atherosclerosis.2014.12.030 25576847

[26] Wang K Basu R Poglitsch M Bakal J Oudit G . Elevated angiotensin 1–7/angiotensin II ratio predicts favorable outcomes in patients with heart failure. Circulation: Heart Failure. (2020) 13:e006939. doi: 10.1161/circheartfailure.120.006939 32580658

[27] Sivakumar R Aravaanan ASK Mohanakrishnan VV Kumar J . Adropin and endothelin-1 as complementary signals associated with early vascular aging in middle-aged type 2 diabetes. Diseases. (2026) 14(4):140. doi: 10.3390/diseases14040140 42041632 PMC13115351

[28] Lillie EO Mahata M Khandrika S Rao F Bundey RA Wen G . Heredity of endothelin secretion: Human twin studies reveal the influence of polymorphism at the chromogranin A locus, a novel determinant of endothelial function. Circulation. (2007) 115:2282–91. doi: 10.1161/circulationaha.106.648345 17438153

[29] Verweij N Mahmud H Mateo Leach I de Boer RA Brouwers FP Yu H . Genome-wide association study on plasma levels of midregional-proadrenomedullin and C-terminal-pro-endothelin-1. Hypertension. (2013) 61:602–8. doi: 10.1161/hypertensionaha.111.203117 23381795

[30] Grzegorzewska A Niepolski L Mostowska A Warchoł W Jagodziński P . Involvement of adropin and adropin-associated genes in metabolic abnormalities of hemodialysis patients. Life Sci. (2016) 160:41–6. doi: 10.1016/j.lfs.2016.07.009 27449397

[31] Grzegorzewska AE Niepolski L Świderska MK Mostowska A Stolarek I Warchoł W . ENHO, RXRA, and LXRA polymorphisms and dyslipidaemia, related comorbidities and survival in haemodialysis patients. BMC Med Genet. (2018) 19(1):194. doi: 10.1186/s12881-018-0708-4 30413149 PMC6234788

[32] Tiret L Poirier O Hallet V McDonagh TA Morrison C McMurray JJV . The Lys198Asn polymorphism in the endothelin-1 gene is associated with blood pressure in overweight people. Hypertension. (1999) 33:1169–74. doi: 10.1161/01.hyp.33.5.1169 10334806

[33] Ameen OM Abbas A . Association of endothelin-1 (EDN1) rs5370 polymorphism and serum ET-1 levels with coronary artery disease and hypertension in Duhok Province population. Egyptian J Internal Med. (2025) 37(1):159. doi: 10.1186/s43162-025-00556-038164791

[34] Fang Z Li M Z Tu G . Association of endothelin-1 gene polymorphisms with essential hypertension in a Chinese population. Genet Mol Res GMR. (2017) 16(3):gmr16037446. doi: 10.4238/gmr16037446 28692114

[35] Mei M Cheng G Sun B Yang L Wang H Sun J . EDN1 gene variant is associated with neonatal persistent pulmonary hypertension. Sci Rep. (2016) 6:29877. doi: 10.1038/srep29877 27425626 PMC4947908

[36] Zhang J Zhang S Qiao J Wang T Zeng P . Similarity and diversity of genetic architecture for complex traits between East Asian and European populations. BMC Genomics. (2023) 24(1):314. doi: 10.1186/s12864-023-09434-x 37308816 PMC10258967

[37] Kumari B Prabhakar A Sahu A Chatterjee T Tyagi T Gupta N . Endothelin-1 gene polymorphism and its level predict the risk of venous thromboembolism in male Indian population. Clin Appl Thrombosis/Hemostasis. (2017) 23:429–37. doi: 10.1177/1076029616661416 27481876

[38] Huels A Ickstadt K Schikowski T Krämer U . Detection of gene-environment interactions in the presence of linkage disequilibrium and noise by using genetic risk scores with internal weights from elastic net regression. BMC Genet. (2017) 18(1):55. doi: 10.1186/s12863-017-0519-1 28606108 PMC5469185

[39] Choi SW García-González J Ruan Y Wu HM Porras C Johnson J . PRSet: Pathway-based polygenic risk score analyses and software. PloS Genet. (2023) 19(2):e1010624. doi: 10.1371/journal.pgen.1010624 36749789 PMC9937466

[40] Lin J Mars N Fu Y Ripatti P Kiiskinen T FinnGen study . Integration of biomarker polygenic risk score improves prediction of coronary heart disease. JACC: Basic to Trans Sci. (2023) 8:1489–99. doi: 10.1016/j.jacbts.2023.07.006 38205343 PMC10774750

[41] Ritchie SC Jiang X Pennells L Xu Y Coffey C Liu Y . Combined clinical, metabolomic, and polygenic scores for cardiovascular risk prediction. Eur Heart J. (2025) 47:1861–73. doi: 10.1093/eurheartj/ehaf947 41392353 PMC13079997

[42] American Diabetes Association Professional Practice Committee . 2. Diagnosis and classification of diabetes: Standards of care in diabetes-2024. Diabetes Care. (2024) 47:S20–42. doi: 10.2337/dc24-S002 38078589 PMC10725812

[43] Politi C Roumeliotis S Tripepi G Spoto B . Sample size calculation in genetic association studies: A practical approach. Life (Basel). (2023) 13:235. doi: 10.3390/life13010235 36676184 PMC9863799

[44] Papaioannou T Protogerou A Vrachatis D Konstantonis G Aissopou E Argyris A . Mean arterial pressure values calculated using seven different methods and their associations with target organ deterioration in a single-center study of 1878 individuals. Hypertens Res. (2016) 39:640–7. doi: 10.1038/hr.2016.41 27194570

[45] Ji C Gao J Huang Z Chen C Wang G Wu S . Estimated pulse wave velocity and cardiovascular events in Chinese. Int J Cardiol Hypertens. (2020) 7:100063. doi: 10.1016/j.ijchy.2020.100063 33447784 PMC7803041

[46] Okonechnikov K Golosova O Fursov M UGENE team . Unipro UGENE: A unified *bioinformatics* toolkit. Bioinformatics. (2012) 28:1166–7. doi: 10.1093/bioinformatics/bts091 22368248

[47] Fujie S Hasegawa N Horii N Uchida M Sanada K Hamaoka T . Aerobic exercise restores aging-associated reductions in arterial adropin levels and improves adropin-induced nitric oxide-dependent vasorelaxation. J Am Heart Assoc. (2021) 10:e020641. doi: 10.1161/JAHA.120.020641 33938228 PMC8200711

[48] Donato AJ Gano LB Eskurza I Silver AE Gates PE Jablonski K . Vascular endothelial dysfunction with aging: Endothelin-1 and endothelial nitric oxide synthase. Am J Physiol Heart Circ Physiol. (2009) 297:H425–32. doi: 10.1152/ajpheart.00689.2008 19465546 PMC2711733

[49] Wu L Fang J Chen L Zhao Z Luo Y Lin C . Low serum adropin is associated with coronary atherosclerosis in type 2 diabetic and non-diabetic patients. Clin Chem Lab Med. (2014) 52:751–8. doi: 10.1515/cclm-2013-0844 24323892

[50] Zheng J Liu M Chen L Yin F Zhu X Gou J . Association between serum adropin level and coronary artery disease: A systematic review and meta-analysis. Cardiovasc Diagn Ther. (2019) 9:1–7. doi: 10.21037/cdt.2018.07.09 30881871 PMC6382666

[51] Zhao ZW Ren YG Liu J . Low serum adropin levels are associated with coronary slow flow phenomenon. Acta Cardiol Sin. (2018) 34:307–12. doi: 10.6515/ACS.201807_34(4).20180306B 30065568 PMC6066951

[52] Maciorkowska M Musiałowska D Małyszko J . Adropin and irisin in arterial hypertension, diabetes mellitus and chronic kidney disease. Adv Clin Exp Med. (2019) 28:1571–5. doi: 10.17219/acem/104551 31756066

[53] Gulen B Eken C Kucukdagli OT Serinken M Kocyigit A Kılıc E . Adropin levels and target organ damage secondary to high blood pressure in the ED. Am J Emerg Med. (2016) 34:2061–4. doi: 10.1016/j.ajem.2016.04.014 27592461

[54] Wiltshire S Powell BL Jennens M McCaskie PA Carter KW Palmer LJ . Investigating the association between K198N coding polymorphism in EDN1 and hypertension, lipoprotein levels, the metabolic syndrome and cardiovascular disease. Hum Genet. (2008) 123:307–13. doi: 10.1007/s00439-008-0481-0 18288492

[55] Oo WM Thiha K Khine MM . Associations of + 138 Ins/del A and + 5665 G/T polymorphisms of endothelin-1 gene with hypertension in Burmese people in Magway, Myanmar. Clin Hypertens. (2022) 28:20. doi: 10.1186/s40885-022-00201-w 35841119 PMC9284899

[56] Tanaka C Kamide K Takiuchi S Kawano Y Miyata T . Evaluation of the Lys198Asn and -134delA genetic polymorphisms of the endothelin-1 gene. Hypertens Res. (2004) 27:367–71. doi: 10.1291/hypres.27.367 15198485

[57] Lago-Docampo M Solarat C Méndez-Martínez L Baloira A Valverde D . Common variation in *EDN1* regulatory regions highlights the role of PPARγ as a key regulator of endothelin *in vitro*. Front Cardiovasc Med. (2022) 9:823133. doi: 10.3389/fcvm.2022.823133 35282351 PMC8913939

[58] Maslat AO Al-Mahmood OM Al Khawaja NM Al-Shdefat R . Association of genetic polymorphisms of *EDN1* gene and endothelin-1 level in patients with type 2 diabetes mellitus in the Jordanian population. Heliyon. (2023) 10:e23676. doi: 10.1016/j.heliyon.2023.e23676 38187330 PMC10767158

[59] Zhang L Sui R . Effect of SNP polymorphisms of EDN1, EDNRA, and EDNRB gene on ischemic stroke. Cell Biochem Biophys. (2014) 70:233–9. doi: 10.1007/s12013-014-9887-6 24633486

[60] Gottsäter M Hindy G Orho-Melander M Nilsson PM Melander O . A genetic risk score for fasting plasma glucose is independently associated with arterial stiffness: a Mendelian randomization study. J Hypertens. (2018) 36:809–14. doi: 10.1097/HJH.0000000000001646 29215398 PMC5861999

[61] Lawlor DA Tilling K Davey Smith G . Triangulation in aetiological epidemiology. Int J Epidemiol. (2016) 45:1866–86. doi: 10.1093/ije/dyw314 28108528 PMC5841843

[62] Maxwell SE Cole DA . Bias in cross-sectional analyses of longitudinal mediation. Psychol Methods. (2007) 12:23–44. doi: 10.1037/1082-989X.12.1.23 17402810

[63] Preacher KJ Kelley K . Effect size measures for mediation models: quantitative strategies for communicating indirect effects. Psychol Methods. (2011) 16:93–115. doi: 10.1037/a0022658 21500915

[64] Pek SLT Lim SC Ang K Kwan PY Tang WE Sum CF . Endothelin-1 predicts incident diabetic peripheral neuropathy in type 2 diabetes: a cohort study. Eur J Endocrinol. (2020) 182:429–38. doi: 10.1530/EJE-19-0523 32061157

[65] O'Sullivan JW Raghavan S Marquez-Luna C Luzum JA Damrauer SM Ashley EA . Polygenic risk scores for cardiovascular disease: a scientific statement from the American Heart Association. Circulation. (2022) 146:e93–e118. doi: 10.1161/CIR.0000000000001077 35862132 PMC9847481

[66] Elliott J Bodinier B Bond TA Chadeau-Hyam M Evangelou E Moons KGM . Predictive accuracy of a polygenic risk score-enhanced prediction model vs a clinical risk score for coronary artery disease. JAMA. (2020) 323:636–45. doi: 10.1001/jama.2019.22241 32068818 PMC7042853

