# Supplementary material for: ENHO and EDN1 variants link adropin–endothelin-1 imbalance with early vascular aging in adults with and without type 2 diabetes
Source: Front Endocrinol (Lausanne). 2026 Oct 6;17:1961984. doi: 10.3389/fendo.2026.1961984 (PMC13680144; doi:10.3389/fendo.2026.1961984)
Supplement: Supplementary file 1 [file Table1.docx]

Supplementary Material

**Supplementary Table S1. Candidate variant selection funnel and evidence-prioritization rationale for ENHO and EDN1 variants**

| Steps | Screening criterion | ENHO retained | EDN1 retained | Total retained | Number excluded/deprioritized | Rationale |
| --- | --- | --- | --- | --- | --- | --- |
| Step 1 | Annotated variants reviewed from public databases | 2,095 | 17,100 | 19,195 | - | Starting candidate-gene variant pool for ENHO and EDN1. |
| Step 2 | Common variants retained using MAF >0.05 | 5 | 238 | 243 | 18,952 | Rare variants were excluded (MAF > 0.05) |
| Step 3 | Merged or deprecated rsIDs resolved to unique current rsIDs | 3 | 98 | 101 | 142 | Merged and deprecated records were collapsed into current rsIDs to avoid duplicate counting of the same variant. |
| Step 4 | Unique SNV-level candidates retained | 3 | 82 | 85 | 16 | Non-SNV, deletion, deletion-insertion, and complex variant records were excluded to retain single-nucleotide candidates. |
| Step 5 | Candidate-priority shortlist applied | 3 | 7 | 10 | 75 | Variants were prioritized based on coding consequence, interpretable intronic or gene-level context, prior database or literature support, and biological relevance to the ENHO/adropin or EDN1/endothelin-1 axis. |
| Step 6 | IndiGenomes Indian-frequency confirmation | 3 | 7 | 10 | 0 | All 10 candidate-priority variants were present in IndiGenomes. Therefore, the Indian-frequency layer was used as population-relevance confirmation. |
| Step 7 | Evidence-prioritized final pathway-balanced panel | 2 | 2 | 4 | 6 | Two ENHO and two EDN1 variants were selected based on evidence strength, variant-class complementarity, and fit with the adropin–endothelin-1 imbalance hypothesis. |

**MAF, minor allele frequency; SNV, single-nucleotide variant. Data retrieved from NCBI dbSNP and IndiGenomes.**

**Supplementary Table S2. Primer sequences and amplicon characteristics**

| Gene | SNP | Nucleotide change | Forward primer | Reverse primer | Product size | Minimum genotype ΔTm | In silico specificity |
| --- | --- | --- | --- | --- | --- | --- | --- |
| ENHO | rs2281997 | T>C \| **C>T** | TCACCCCACCCTTCCTCCTCAC | CCTGGGCAGTGGAGATGTCTACC | 134 bp | 0.46°C | BLAST pass |
| ENHO | rs72735260 | G>T | CTGGACTATGCTCCATCCCAAGGG | TTCCCCAGCCTAGAGGGCCTAG | 138 bp | 0.45°C | BLAST pass |
| EDN1 | rs5370 | G>T | CGGAGACCATGAGAAACAGCGTCA | TGCTCGGTTGTGGGTCACATAACG | 143 bp | 0.43°C | BLAST pass |
| EDN1 | rs2070699 | G>T | GGTCAACACTCCCGAGTAAGTCTC | CTCTAAAACTGGGCTCCAGTGGAG | 134 bp | 0.48°C | BLAST pass |

**ΔTm, difference in melting temperature between homozygous genotype classes; F, forward primer; R, reverse primer; NCBI BLAST was performed to check primer specificity; NCBI reference allele for rs2281997 is C; however, T allele is reported as minor/ Risk allele in SAS and in several studies.**

**Supplementary Table S3. Full inheritance-model associations of ENHO and EDN1 variants with early vascular aging**

| Gene | SNP | Model | Contrast | Crude OR (95% CI) | Crude *p* | Crude FDR | Crude AIC | Adjusted OR (95% CI) | Adjusted *p* | Adjusted FDR | Adjusted AIC | AIC-selected |
| --- | --- | --- | --- | --- | --- | --- | --- | --- | --- | --- | --- | --- |
| ENHO | rs2281997 | Codominant | C/T vs C/C | 1.803 (1.024-3.177) | 0.0408 | 0.0816 | 401 | 1.797 (1.019-3.174) | 0.0425 | 0.0851 | 402.99 | Dominant |
| ENHO | rs2281997 | Codominant | T/T vs C/C | 2.233 (0.653-7.967) | 0.196 | 0.235 | 401 | 2.234 (0.653-7.971) | 0.196 | 0.235 | 402.99 | Dominant |
| ENHO | rs2281997 | Dominant | C/T + T/T vs C/C | 1.861 (1.093-3.173) | 0.022 | 0.0728 | 399.11 | 1.857 (1.089-3.171) | 0.0228 | 0.075 | 401.1 | Dominant |
| ENHO | rs2281997 | Recessive | T/T vs C/C + C/T | 1.953 (0.575-6.919) | 0.278 | 0.278 | 403.16 | 1.958 (0.577-6.941) | 0.277 | 0.277 | 405.08 | Dominant |
| ENHO | rs2281997 | Over-dominant | C/T vs C/C + T/T | 1.732 (0.987-3.041) | 0.0548 | 0.0822 | 400.67 | 1.728 (0.983-3.039) | 0.0569 | 0.0854 | 402.66 | Dominant |
| ENHO | rs2281997 | Log-additive | Per T allele | 1.655 (1.07-2.583) | 0.0243 | 0.0728 | 399.22 | 1.652 (1.067-2.58) | 0.025 | 0.075 | 401.2 | Dominant |
| ENHO | rs72735260 | Codominant | G/T vs G/G | 2.025 (1.148-3.587) | 0.0148 | 0.0297 | 399.33 | 2.034 (1.149-3.616) | 0.0149 | 0.0297 | 401.31 | Dominant |
| ENHO | rs72735260 | Codominant | T/T vs G/G | 2.373 (0.612-9.824) | 0.207 | 0.248 | 399.33 | 2.408 (0.611-10.117) | 0.205 | 0.246 | 401.31 | Dominant |
| ENHO | rs72735260 | Dominant | G/T + T/T vs G/G | 2.066 (1.206-3.556) | 0.00837 | 0.0297 | 397.38 | 2.076 (1.204-3.596) | 0.00871 | 0.0297 | 399.37 | Dominant |
| ENHO | rs72735260 | Recessive | T/T vs G/G + G/T | 2.027 (0.526-8.34) | 0.3 | 0.3 | 403.26 | 2.002 (0.512-8.336) | 0.314 | 0.314 | 405.24 | Dominant |
| ENHO | rs72735260 | Over-dominant | G/T vs G/G + T/T | 1.956 (1.112-3.452) | 0.0199 | 0.0299 | 398.93 | 1.952 (1.107-3.455) | 0.0208 | 0.0311 | 400.92 | Dominant |
| ENHO | rs72735260 | Log-additive | Per T allele | 1.815 (1.152-2.895) | 0.0109 | 0.0297 | 397.74 | 1.827 (1.152-2.934) | 0.0111 | 0.0297 | 399.71 | Dominant |
| EDN1 | rs5370 | Codominant | G/T vs G/G | 1.835 (1.098-3.088) | 0.0211 | 0.0254 | 395.63 | 1.863 (1.111-3.145) | 0.0188 | 0.0246 | 397.3 | Log-additive |
| EDN1 | rs5370 | Codominant | T/T vs G/G | 2.88 (1.437-5.846) | 0.003 | 0.00601 | 395.63 | 2.92 (1.454-5.942) | 0.00274 | 0.00547 | 397.3 | Log-additive |
| EDN1 | rs5370 | Dominant | G/T + T/T vs G/G | 2.073 (1.288-3.369) | 0.00291 | 0.00601 | 395.25 | 2.104 (1.304-3.431) | 0.00255 | 0.00547 | 396.91 | Log-additive |
| EDN1 | rs5370 | Recessive | T/T vs G/G + G/T | 2.139 (1.123-4.116) | 0.0211 | 0.0254 | 399 | 2.147 (1.127-4.134) | 0.0205 | 0.0246 | 400.88 | Log-additive |
| EDN1 | rs5370 | Over-dominant | G/T vs G/G + T/T | 1.386 (0.863-2.226) | 0.176 | 0.176 | 402.51 | 1.397 (0.869-2.247) | 0.168 | 0.168 | 404.36 | Log-additive |
| EDN1 | rs5370 | Log-additive | Per T allele | 1.726 (1.242-2.417) | 0.00129 | 0.00601 | 393.72 | 1.74 (1.25-2.442) | 0.00115 | 0.00547 | 395.41 | Log-additive |
| EDN1 | rs2070699 | Codominant | G/T vs G/G | 1.147 (0.691-1.906) | 0.594 | 0.713 | 400.27 | 1.147 (0.691-1.905) | 0.595 | 0.715 | 402.26 | Recessive |
| EDN1 | rs2070699 | Codominant | T/T vs G/G | 2.453 (1.196-5.116) | 0.015 | 0.05 | 400.27 | 2.467 (1.193-5.187) | 0.0155 | 0.0519 | 402.26 | Recessive |
| EDN1 | rs2070699 | Dominant | G/T + T/T vs G/G | 1.386 (0.869-2.223) | 0.172 | 0.259 | 402.47 | 1.383 (0.866-2.219) | 0.176 | 0.263 | 404.42 | Recessive |
| EDN1 | rs2070699 | Recessive | T/T vs G/G + G/T | 2.299 (1.167-4.601) | 0.0167 | 0.05 | 398.55 | 2.313 (1.164-4.672) | 0.0173 | 0.0519 | 400.54 | Recessive |
| EDN1 | rs2070699 | Over-dominant | G/T vs G/G + T/T | 0.934 (0.58-1.498) | 0.777 | 0.777 | 404.26 | 0.938 (0.582-1.506) | 0.79 | 0.79 | 406.19 | Recessive |
| EDN1 | rs2070699 | Log-additive | Per T allele | 1.444 (1.034-2.026) | 0.0316 | 0.0632 | 399.69 | 1.443 (1.031-2.028) | 0.033 | 0.066 | 401.68 | Recessive |

**Supplementary Table S4. Individual SNP associations with continuous ePWV**

| Gene | SNP | Model | Genotype contrast | Beta 95 CI | *p* value | FDR *p*-value | AIC |
| --- | --- | --- | --- | --- | --- | --- | --- |
| ENHO | rs2281997 | Codominant | C/T vs C/C | 0.235 (0.015 to 0.456) | 0.03660 | 0.0672 | 710.75 |
| ENHO | rs2281997 | Codominant | T/T vs C/C | 0.232 (-0.244 to 0.708) | 0.33900 | 0.4060 | 710.75 |
| ENHO | rs2281997 | Dominant | C/T + T/T vs C/C | 0.235 (0.028 to 0.441) | 0.02620 | 0.0672 | 708.75 |
| ENHO | rs2281997 | Recessive | T/T vs C/C + C/T | 0.181 (-0.295 to 0.657) | 0.45500 | 0.4550 | 713.19 |
| ENHO | rs2281997 | Over-dominant | C/T vs C/C + T/T | 0.224 (0.005 to 0.444) | 0.04480 | 0.0672 | 709.68 |
| ENHO | rs2281997 | Log-additive | Per T allele | 0.181 (0.011 to 0.35) | 0.03680 | 0.0672 | 709.34 |
| ENHO | rs72735260 | Codominant | G/T vs G/G | 0.232 (0.011 to 0.453) | 0.04000 | 0.0781 | 708.78 |
| ENHO | rs72735260 | Codominant | T/T vs G/G | 0.497 (-0.031 to 1.025) | 0.06500 | 0.0781 | 708.78 |
| ENHO | rs72735260 | Dominant | G/T + T/T vs G/G | 0.264 (0.053 to 0.475) | 0.01430 | 0.0429 | 707.68 |
| ENHO | rs72735260 | Recessive | T/T vs G/G + G/T | 0.439 (-0.089 to 0.967) | 0.10300 | 0.1030 | 711.06 |
| ENHO | rs72735260 | Over-dominant | G/T vs G/G + T/T | 0.21 (-0.011 to 0.431) | 0.06210 | 0.0781 | 710.23 |
| ENHO | rs72735260 | Log-additive | Per T allele | 0.239 (0.061 to 0.416) | 0.00869 | 0.0429 | 706.79 |
| EDN1 | rs5370 | Codominant | G/T vs G/G | 0.01 (-0.184 to 0.204) | 0.91900 | 0.9190 | 711.91 |
| EDN1 | rs5370 | Codominant | T/T vs G/G | 0.255 (-0.014 to 0.523) | 0.06280 | 0.1880 | 711.91 |
| EDN1 | rs5370 | Dominant | G/T + T/T vs G/G | 0.076 (-0.105 to 0.256) | 0.40900 | 0.6140 | 713.07 |
| EDN1 | rs5370 | Recessive | T/T vs G/G + G/T | 0.25 (-0.002 to 0.502) | 0.05170 | 0.1880 | 709.92 |
| EDN1 | rs5370 | Over-dominant | G/T vs G/G + T/T | -0.053 (-0.236 to 0.13) | 0.57000 | 0.6840 | 713.43 |
| EDN1 | rs5370 | Log-additive | Per T allele | 0.099 (-0.027 to 0.225) | 0.12200 | 0.2440 | 711.33 |
| EDN1 | rs2070699 | Codominant | G/T vs G/G | 0.189 (-0.002 to 0.379) | 0.05280 | 0.0793 | 709.47 |
| EDN1 | rs2070699 | Codominant | T/T vs G/G | 0.302 (0.02 to 0.583) | 0.03580 | 0.0716 | 709.47 |
| EDN1 | rs2070699 | Dominant | G/T + T/T vs G/G | 0.216 (0.037 to 0.394) | 0.01790 | 0.0537 | 708.08 |
| EDN1 | rs2070699 | Recessive | T/T vs G/G + G/T | 0.214 (-0.054 to 0.483) | 0.11700 | 0.1410 | 711.27 |
| EDN1 | rs2070699 | Over-dominant | G/T vs G/G + T/T | 0.124 (-0.058 to 0.306) | 0.18100 | 0.1810 | 711.94 |
| EDN1 | rs2070699 | Log-additive | Per T allele | 0.162 (0.033 to 0.29) | 0.01370 | 0.0537 | 707.61 |

**Supplementary Table S5. LD matrix, haplotype frequencies, and haplotype associations for ENHO and EDN1 variants**

| Analysis | Gene | SNP pair/order | Haplotype | Frequency | D′ | r² | Global *p* | LD/haplotype *p* |
| --- | --- | --- | --- | --- | --- | --- | --- | --- |
| Pairwise LD | ENHO | rs2281997–rs72735260 | - | - | 0.251 | 0.0016 | - | 0.326 |
|  | EDN1 | rs5370–rs2070699 | - | - | 0.0077 | <0.001 | - | 0.922 |
| Haplotype association | ENHO | rs2281997–rs72735260 | C–G | 0.7391 | - | - | 0.060 | 0.0097 |
|  | ENHO | rs2281997–rs72735260 | T–G | 0.1275 | - | - | 0.060 | 0.079 |
|  | ENHO | rs2281997–rs72735260 | C–T | 0.1192 | - | - | 0.060 | 0.089 |
|  | ENHO | rs2281997–rs72735260 | T–T | 0.0141 | - | - | 0.060 | 0.880 |
|  | EDN1 | rs5370–rs2070699 | G–G | 0.4347 | - | - | 0.025 | 0.052 |
|  | EDN1 | rs5370–rs2070699 | T–G | 0.2320 | - | - | 0.025 | 0.0043 |
|  | EDN1 | rs5370–rs2070699 | G–T | 0.2187 | - | - | 0.025 | - |
|  | EDN1 | rs5370–rs2070699 | T–T | 0.1147 | - | - | 0.025 | - |
| *D′ and r² were estimated for each within-gene variant pair in the full cohort. Haplotype alleles are presented in the SNP order shown in the table. Global p values assess the overall haplotype association with EVA, whereas haplotype-specific p values represent individual estimated haplotype effects.* | | | | | | | | |

**Supplementary Table S6. Genotype-stratified adropin, ET-1, and AEI levels with adjusted trend tests**

| Gene | SNP | Biomarker | Risk allele | Major homozygote | Heterozygote | Minor homozygote | Trend β (95% CI) | Trend *p* | FDR *p* |
| --- | --- | --- | --- | --- | --- | --- | --- | --- | --- |
| *ENHO* | rs2281997 | ln(adropin) | T | C/C: 6.519 (5.546–7.206) | C/T: 5.520 (4.606–6.140) | T/T: 5.219 (4.865–5.585) | −0.739 (−0.943 to −0.535) | <0.001 | <0.001 |
| *ENHO* | rs2281997 | ln(ET-1) | T | C/C: 0.604 (0.249–1.674) | C/T: 1.182 (0.278–1.823) | T/T: 1.681 (1.031–1.960) | 0.283 (0.063 to 0.503) | 0.012 | 0.016 |
| *ENHO* | rs2281997 | AEI | T | C/C: −0.710 (−1.794–1.087) | C/T: 0.900 (−0.668–1.787) | T/T: 0.980 (0.180–2.514) | 0.997 (0.635 to 1.360) | <0.001 | <0.001 |
| *ENHO* | rs72735260 | ln(adropin) | T | G/G: 6.366 (5.428–6.995) | G/T: 5.667 (5.365–6.352) | T/T: 5.429 (5.369–6.080) | −0.359 (−0.589 to −0.130) | 0.002 | 0.004 |
| *ENHO* | rs72735260 | ln(ET-1) | T | G/G: 0.683 (0.262–1.675) | G/T: 1.166 (0.258–1.849) | T/T: 1.157 (0.588–1.587) | 0.161 (−0.073 to 0.394) | 0.177 | 0.177 |
| *ENHO* | rs72735260 | AEI | T | G/G: −0.560 (−1.647–1.091) | G/T: 0.156 (−0.956–1.864) | T/T: 0.166 (0.000–1.200) | 0.508 (0.112 to 0.904) | 0.012 | 0.013 |
| *EDN1* | rs5370 | ln(adropin) | T | G/G: 6.272 (5.447–6.839) | G/T: 6.216 (5.172–7.120) | T/T: 5.722 (5.377–6.716) | −0.090 (−0.253 to 0.074) | 0.281 | 0.281 |
| *EDN1* | rs5370 | ln(ET-1) | T | G/G: 0.670 (0.255–1.562) | G/T: 0.392 (0.231–1.722) | T/T: 1.649 (0.589–1.913) | 0.272 (0.110 to 0.434) | 0.001 | 0.002 |
| *EDN1* | rs5370 | AEI | T | G/G: −0.604 (−1.384–1.028) | G/T: −0.303 (−1.704–1.424) | T/T: 0.988 (−1.001–1.783) | 0.353 (0.075 to 0.632) | 0.013 | 0.013 |
| *EDN1* | rs2070699 | ln(adropin) | T | G/G: 6.366 (5.446–6.843) | G/T: 6.217 (5.467–6.904) | T/T: 5.451 (4.715–6.290) | −0.217 (−0.383 to −0.05) | 0.011 | 0.014 |
| *EDN1* | rs2070699 | ln(ET-1) | T | G/G: 0.658 (0.293–1.302) | G/T: 0.565 (0.201–1.796) | T/T: 1.589 (0.389–1.974) | 0.330 (0.165 to 0.495) | <0.001 | <0.001 |
| *EDN1* | rs2070699 | AEI | T | G/G: −0.560 (−1.395–0.811) | G/T: −0.368 (−1.640–1.411) | T/T: 1.091 (−0.767–2.590) | 0.534 (0.251 to 0.816) | <0.001 | <0.001 |

**Supplementary Table S7. Full mediation output and sensitivity models**

| Model | Exposure | Mediator | Outcome | ACME 95CI | ADE 95CI | ADE *p* | Total effect 95CI | Total *p* | Proportion mediated 95CI | Prop *p* |
| --- | --- | --- | --- | --- | --- | --- | --- | --- | --- | --- |
| Primary mediation | Total GRS | AEI | EVA | 0.047 (0.028 to 0.069) | 0.039 (0.009 to 0.064) | 0.0144 | 0.091 (0.062 to 0.114) | <0.001 | 0.518 (0.305 to 0.868) | <0.001 |
| Robustness mediation | Total GRS | AEI | ePWV | 0.113 (0.079 to 0.149) | 0.041 (-0.025 to 0.105) | 0.2140 | 0.153 (0.081 to 0.223) | <0.001 | 0.735 (0.49 to 1.283) | <0.001 |
| ENHO-specific mediation | ENHO-GRS | ln(adropin) | EVA | 0.117 (0.074 to 0.163) | 0.02 (-0.049 to 0.099) | 0.5930 | 0.139 (0.062 to 0.223) | <0.001 | 0.84 (0.465 to 1.768) | <0.001 |
| ENHO-specific mediation | ENHO-GRS | ln(adropin) | ePWV | 0.234 (0.168 to 0.308) | -0.014 (-0.14 to 0.108) | 0.8350 | 0.22 (0.084 to 0.353) | 0.0016 | 1.064 (0.665 to 2.48) | 0.0016 |
| EDN1-specific mediation | EDN1-GRS | ln(ET-1) | EVA | 0.043 (0.024 to 0.065) | 0.051 (0.008 to 0.089) | 0.0288 | 0.098 (0.05 to 0.138) | <0.001 | 0.437 (0.239 to 0.86) | <0.001 |
| EDN1-specific mediation | EDN1-GRS | ln(ET-1) | ePWV | 0.076 (0.044 to 0.111) | 0.053 (-0.029 to 0.138) | 0.2070 | 0.129 (0.041 to 0.214) | 0.0028 | 0.586 (0.309 to 1.646) | 0.0028 |

**Supplementary Table S8. ROC analysis for early vascular aging discrimination**

| Model | AUC (95% CI) | Youden threshold | Sensitivity | Specificity |
| --- | --- | --- | --- | --- |
| Clinical model | 0.590 (0.523–0.657) | 0.383 | 0.534 | 0.679 |
| Total GRS | 0.661 (0.600–0.722) | 0.332 | 0.750 | 0.489 |
| AEI | 0.776 (0.720–0.833) | 0.382 | 0.741 | 0.761 |
| Clinical + Total GRS | 0.686 (0.624–0.748) | 0.366 | 0.672 | 0.647 |
| Clinical + AEI | 0.776 (0.719–0.832) | 0.414 | 0.716 | 0.777 |
| Clinical + Total GRS + AEI | 0.779 (0.721–0.836) | 0.379 | 0.733 | 0.745 |
| *The clinical model included age, sex, and BMI.* | | | | |

**Figure S1. High-resolution melting genotyping of the four *ENHO* and *EDN1* variants with bidirectional Sanger sequencing confirmation.**

Genotype discrimination for (A) *ENHO* rs2281997 (C>T), (B) *ENHO* rs72735260 (G>T), (C) *EDN1* rs5370 (G>T, Lys198Asn), and (D) *EDN1* rs2070699 (G>T). Within each panel, (i) derivative melt plots (−dF/dT against temperature), (ii) normalized melt curves expressed as percentage helicity, and (iii) difference plots computed relative to the mean major-homozygote curve are shown as individual replicate traces (left) and as genotype mean curves (right). For each variant, samples per genotype were assayed in five replicates, and the mean of these replicates was used to confirm reproducibility of the melting pattern. R = A/G, Y = C/T, K = G/T, and M = A/C.

**Supplementary Figure S2: Pairwise linkage disequilibrium and haplotype distributions for *ENHO* and *EDN1*.**

**Supplementary Figure S3: In silico annotation and cohort-concordance heatmap**
